# *GEfetch2R*: fetching single-cell/bulk RNA-seq data from public repositories to R and benchmarking the subsequent format conversion tools

**DOI:** 10.1093/gigascience/giag039

**Published:** 2026-04-08

**Authors:** Yabing Song, Jianbin Wang, Jiaxin Gao

**Affiliations:** State Key Laboratory of Microbial Diversity and Innovative Utilization, Institute of Microbiology, Chinese Academy of Sciences, Beijing 100101, China; School of Life Sciences, Tsinghua University, Beijing 100084, China; State Key Laboratory of Microbial Diversity and Innovative Utilization, Institute of Microbiology, Chinese Academy of Sciences, Beijing 100101, China

**Keywords:** single-cell/bulk RNA-seq, data download, format conversion, tool benchmark, software

## Abstract

**Background:**

Downloading and reanalyzing the existing single-cell RNA sequencing (scRNA-seq) data provides an efficient choice to gain clues and new insights. However, no tool can fetch the diverse scRNA-seq data types (raw data, count matrix, and processed object) distributed in various repositories, process and load the downloaded data to R, convert formats between scRNA-seq objects, and benchmark the format conversion tools.

**Findings:**

Here, we present *GEfetch2R*, an R package with Docker image to (i) download diverse scRNA-seq data types, including raw data (SRA and ENA), count matrix (GEO, UCSC Cell Browser, and PanglaoDB), and processed objects (GEO, Zenodo, CELLxGENE, and HCA); (ii) process the downloaded data, load the count matrices/annotations/*rds* files to R (*SeuratObject*/*DESeqDataSet*), filter the *SeuratObject* based on cell metadata and genes, and dissect and extract the *RData* files; and (iii) convert formats between the widely used scRNA-seq objects, including *SeuratObject, AnnData, SingleCellExperiment, CellDataSet*/*cell_data_set*, and *loom*, and benchmark format conversion tools in terms of information kept, usability, running time, and scalability to guide the tool selection. Furthermore, *GEfetch2R* can also download, process, and load bulk RNA-seq raw data (SRA and ENA) and count matrices (GEO) to R (*DESeqDataSet*).

**Conclusions:**

*GEfetch2R* is an R package that facilitates researchers in accessing and exploring existing gene expression data from various public repositories. It can function as a data downloader (supports all 3 scRNA-seq and 2 bulk RNA-seq data types), a data processor (processes and loads the output/downloaded count matrices and annotations to R), and an object format converter (between the widely used scRNA-seq objects).

## Introduction

In recent years, single-cell RNA sequencing (scRNA-seq) has emerged as a powerful tool to reveal cellular heterogeneity and fundamental characteristics of gene expression [1]. As a result, thousands of scRNA-seq datasets have been generated and uploaded to public repositories. Downloading and reanalyzing the existing scRNA-seq data provides an efficient choice to gain clues and new insights. For example, rare cell type identification [2] and pan-cancer scRNA-seq analysis [3] usually require a large number of samples and cells, which is extremely expensive and time-consuming unless integrating available scRNA-seq datasets. In addition, exploring existing relevant scRNA-seq data can accelerate the progress of one’s own research.

The existing scRNA-seq data are in 3 types: raw data (*sra*/*fastq*/*bam*), count matrix, and processed object (e.g., *rds* and *h5ad*), which are stored in different repositories. The raw data are mainly stored in Sequence Read Archive (SRA) [4] and European Nucleotide Archive (ENA) [5], the count matrices can be found in Gene Expression Omnibus (GEO) [6] and some scRNA-seq databases (e.g., UCSC Cell Browser [7] and PanglaoDB [8]), and most processed objects are provided by GEO, Zenodo [9], Human Cell Atlas (HCA) [10], and CELLxGENE [11]. The diversity of data types and their distribution across specialized repositories make scRNA-seq data download difficult. Second, the wide array of available scRNA-seq protocols and preprocessing tools has led to differences in the data they produce and store. Representatively, there are differences between 10x Genomics (10x) [12] and Smart-seq2 [13] in terms of sequencing reads and output count matrix forms. How to process and load these different types of downloaded files into a unified object that can be analyzed matters. Third, the formats and programming languages of downloaded processed objects are varied due to the prosperity in scRNA-seq analysis tools, such as *SeuratObject* (*Seurat*, R) [14], *AnnData* (*Scanpy*, Python) [15], *CellDataSet*/*cell_data_set* (*Monocle2*/*3*, R) [16, 17], and *SingleCellExperiment* (*scater*, R) [18]. Diverse object formats and programming languages hinder the integration of scRNA-seq data and the interoperability between different analysis tools; thus, object format conversion is vital.

Currently available tools mainly focus on downloading raw data (SRA and ENA) and count matrices (GEO) (Table 1). *ffq* [19] is designed to fetch sample metadata, including the download links, and therefore requires third-party tools to perform downloading. *GEOfastq* [20], *SRA-Toolkit* [21], *enaBrowserTools* [22], *fastq-dl* [23], *pysradb* [24], and *GEOfetch* [25] support downloading raw data from SRA and/or ENA. Among them, *GEOfastq, fastq-dl*, and *GEOfetch* can only download *fastq, sra*/*fastq*, and *sra* files, respectively, and *GEOfetch* supports splitting *sra* into *fastq*/*bam* files. *GEOfastq, enaBrowserTools, fastq-dl* (version <1.2.0), and *pysradb* support downloading data via Aspera, while *fastq-dl* and *pysradb* parallelize the download process. *pysradb, GEOfetch*, and *GEOquery* [26] can download the supplementary files from GEO, and *GEOquery* can extract the count matrix from *ExpressionSet. rPanglaoDB* [27] can be used to download scRNA-seq count matrices and annotations from PanglaoDB, which only contains datasets originating from human and mouse, has relatively small sample numbers, and is no longer maintained. The above generalized tools, except *rPanglaoDB*, support downloading raw data and/or count matrices from limited databases. This download applies regardless of whether the data are from scRNA-seq or bulk RNA-seq and thus lacks support for unique characteristics of scRNA-seq data (such as 10x-style *fastq* files and *bam* files with custom tags). *rPanglaoDB* specializes in downloading scRNA-seq count matrices and annotations, but the support is quite insufficient. As for format conversion, many tools have been developed for the same conversion stream. From *SeuratObject* to *AnnData* (Seu2AD), available tools are *SeuratDisk, sceasy*, and *scDIOR*, while the above tools and *schard* are suitable for the reverse stream (AD2Seu). From *SingleCellExperiment* to *AnnData* (SCE2AD), available tools are *sceasy, scDIOR*, and *zellkonverter*, while *scDIOR, zellkonverter*, and *schard* are suitable for the reverse stream (AD2SCE). Multiple available tools for the same stream have confused the format conversion process, and there is currently a lack of a benchmark of format conversion tools to guide tool selection. Besides, no single tool can cover all the conversion streams between the widely used scRNA-seq objects.

**Table 1:** Comparison of *GEfetch2R* with other similar tools.

	*GEfetch2R*	*ffq*	*GEOfastq*	*SRA-Toolkit*	*enaBrowser Tools*	*fastq-dl*	*pysradb*	*GEOfetch*	*GEOquery*	*rPanglaoDB*
**Programming language**	R	Python	R	C	Python	Python	Python	Python	R	R
**Accepted accession**	GEO	SRA, ENA, GEO	GEO	SRA, ENA	SRA, ENA	SRA, ENA	SRA, ENA, GEO	GEO	GEO	–
**Supported database**										
Raw data (*sra*/*fastq*/*bam*)	SRA, ENA	–	ENA	SRA	ENA	SRA, ENA	SRA, ENA	SRA	–	–
Count matrix	GEO, PanglaoDB, UCSC Cell Browser	–	–	–	–	–	GEO	GEO	GEO	PanglaoDB
Processed object	GEO, Zenodo, CELLxGENE, Human Cell Atlas	–	–	–	–	–	–	–	–	–
**Downloaded data**										
Metadata	+	+	+	–	+	+	+	+	+	+
Raw data (*sra*/*fastq*/*bam*)	+++	–	*fastq*	+++	+++	*sra, fastq*	+++	*sra*	–	–
Count matrix	+++	–	–	–	–	–	+	+	+	+
Processed object	+++	–	–	–	–	–	–	–	–	–
**Aspera support**	Yes	–	Yes	–	Yes	–^a^	Yes	–	–	–
**Parallel download**	Yes	–	–	–	–	Yes	Yes	–	–	–
**Process downloaded data**										
*sra* to *fastq*	*fastq-dump, fasterq-dump, parallel-fastq-dump* ^b^	–	–	*fastq-dump, fasterq-dump*	–	*fasterq-dump*	–	*fasterq-dump*	–	–
*sra* to *bam*	*sam-dump*	–	–	*sam-dump*	–	–	–	*sam-dump*	–	–
*bam* to *fastq*	*bamtofastq* (10x)									
	*samtools* (others)	–	–	–	–	–	–	–	–	–
format *fastq* (10x)	Yes	–	–	–	–	–	–	–	–	–
mapping	*CellRanger, STAR*	–	–	–	–	–	–	–	–	–
**Load to R**										
*DESeq2*	Yes	–	–	–	–	–	–	–	*MAList, ExpressionSet*	–
*Seurat*	Yes	–	–	–	–	–	–	–	–	Yes
merge *SeuratObjects*	Yes	–	–	–	–	–	–	–	–	Yes

a *fastq-dl* old version (<1.2.0) supports Aspera.

b Parameters optimized for 10x Genomics data.

Symbols used: ‘-’ for absent; ‘+’ for a more quantitative assessment (more ‘+’ indicating better support).

To address the above issues, we present *GEfetch2R*, an R package to (i) fetch scRNA-seq/bulk RNA-seq raw data, count matrices, and processed objects from diverse public repositories; (ii) process the downloaded data, load the count matrices/annotations/*rds* files to R (*SeuratObject*/*DESeqDataSet*), filter the *SeuratObject* based on cell metadata and genes, and dissect and extract the *RData* files; and (iii) convert formats between the widely used scRNA-seq objects and benchmark format conversion tools to guide the tool selection.

## Materials and Methods

### Download, process, and load raw data

With a given GEO accession as input, *GEfetch2R* can download raw data (*sra*/*fastq*/*bam*) from SRA and ENA (Supplementary Fig. S1). First, *GEfetch2R* extracts all sample metadata under the given GEO accession; users can skip this step and provide a data frame containing the interested samples. The output sample metadata are used as input for subsequent raw data download. For *sra* files, *GEfetch2R* uses the *prefetch* command to download them from SRA and uses *ascp, download.file*, and *wget* commands in parallel to download them from ENA.

For *fastq* files in SRA, *GEfetch2R* uses *parallel-fastq-dump* (parallel), *fasterq-dump* (parallel), and *fastq-dump* commands to split the downloaded *sra* files into *fastq* files. If the data are from 10x, the *–split-files* parameter is added, and at least 2 *fastq* files are generated. *GEfetch2R* automatically distinguishes read1 and read2 based on read length and renames them according to the *CellRanger* required format. As for data from other scRNA-seq protocols or bulk RNA-seq, the *–split-3* parameter is used. For *fastq* files in ENA, *GEfetch2R* supports splitting the downloaded *sra* files as above; alternatively, if there are *fastq* files in ENA, *GEfetch2R* uses *ascp, download.file*, and *wget* commands in parallel to download them directly.

For *bam* files in SRA, if they are from 10x, *GEfetch2R* uses the *prefetch* command with the *–type TenX* parameter to download the original uploaded *bam* files directly to keep the custom tags required to reconstruct the original *fastq* files. As for *bam* files from other scRNA-seq protocols or bulk RNA-seq, *GEfetch2R* uses *sam-dump* commands to split the downloaded *sra* files into *bam* files. For *bam* files in ENA, *GEfetch2R* uses *ascp, download.file*, and *wget* commands in parallel to download them directly. If *fastq* files are needed, *GEfetch2R* uses *bamtofastq* command developed by 10x to convert the downloaded 10x-generated *bam* files to *fastq* files, as well as the *samtools* [28] command to convert the downloaded *bam* files generated by other scRNA-seq protocols or bulk RNA-seq to *fastq* files.

With prepared *fastq* files, if they are from 10x, *GEfetch2R* uses *CellRanger* to perform read alignment and feature counting (merge multiple runs of a sample). The output count matrix is then loaded to R using *Seurat*. If multiple count matrices are available, *GEfetch2R* merges multiple *SeuratObjects* if applicable. As for *fastq* files from Smart-seq2 or bulk RNA-seq, *GEfetch2R* uses *STAR* to perform read alignment and feature counting with the *–quantMode GeneCounts* parameter (merge multiple runs of a sample). The output count matrix is then loaded to R using *DESeq2*.

To simplify the procedures from downloading raw data to loading to R, *GEfetch2R* provides a wrapper function *DownloadFastq2R*, which can extract all runs under a given GEO accession, automatically identify the RNA-seq type (10x Genomics scRNA-seq, Smart-seq2 scRNA-seq, and bulk RNA-seq) of each run, download *fastq* files directly from ENA, perform read alignment and feature counting using *CellRanger*/*STAR* (merge multiple runs of a sample), and load the results to R (*SeuratObject*/*DESeqDataSet*).

### Download, process, and load count matrices and annotations

As in Supplementary Fig. S1, *GEfetch2R* provides 2 ways to access count matrices: with given accessions or links as input (GEO, UCSC Cell Browser, and PanglaoDB) and with filtered metadata as input (UCSC Cell Browser and PanglaoDB). The first way can be used to download specific datasets. For the latter way, *GEfetch2R* first extracts detailed metadata of all datasets, such as description, source/tissue, organism, protocol, and related publication. Users can filter datasets based on these attributes, and the filtered metadata are used as subsequent input. This way can be used to download datasets with similar characteristics in bulk.

When fetching count matrices from GEO, *GEfetch2R* tries to extract the count matrix from *ExpressionSet* first. If the extracted count matrix is empty or contains noninteger values, *GEfetch2R* generates the count matrix from supplementary files. For supplementary files in the *CellRanger* output format, *GEfetch2R* automatically categorizes the downloaded files based on sample names. For supplementary files composed of the count matrix of every single-cell/bulk sample, *GEfetch2R* creates a count matrix containing all cells/samples. Users can also add the *down.supp = TRUE* parameter in *ParseGEO* function to generate the count matrix directly from supplementary files. Besides, *GEfetch2R* also provides function *ExtractGEOMeta* to retrieve detailed sample metadata from the supplementary file or the GEO platform (user-provided when uploading). For the UCSC Cell Browser, *GEfetch2R* accesses count matrices online instead of downloading them to save time and disk usage. In addition to count matrices, the UCSC Cell Browser contains diverse annotations, such as cell-type annotation, cell-type composition, and dimensionality reduction coordinates; *GEfetch2R* supports extracting all these annotations. PanglaoDB, similar to the UCSC Cell Browser, also contains various annotations (e.g., cell-type annotation and cell-type composition), and *GEfetch2R* uses *rPanglaoDB* to extract count matrices and corresponding annotations.

With available scRNA-seq count matrices and various annotations, *GEfetch2R* loads the count matrices to R using *Seurat*, adds available annotations to the *SeuratObjects* (UCSC Cell Browser and PanglaoDB), filters the *SeuratObjects* based on cell metadata and genes (UCSC Cell Browser and PanglaoDB), and merges *SeuratObjects* if applicable. For the bulk RNA-seq count matrix containing all samples, *GEfetch2R* loads it to R using *DESeq2* (GEO).

### Download and load processed objects

Similar to downloading count matrices, *GEfetch2R* also provides 2 ways to access processed objects: with given accessions or links as input (GEO, Zenodo, CELLxGENE, and HCA) and with filtered metadata as input (CELLxGENE and HCA) (Supplementary Fig. S1).

For processed objects in GEO/Zenodo, with provided accession/DOIs, the index of the supplementary file (GEO), and the file extensions (e.g., *rds* and *h5ad*), *GEfetch2R* downloads the corresponding processed objects. For processed objects in CELLxGENE, *GEfetch2R* provides 2 methods to download them. *GEfetch2R* uses *cellxgene-census* [29] developed by the CELLxGENE team to efficiently access the cloud-hosted Census single-cell data and filter the data based on cell metadata and genes. However, some newly uploaded data that can be explored on the webpage may be missing due to delays in cloud hosting (e.g., the stable Census release is 20 January 2025, while the current date is 26 February 2025). To overcome this, *GEfetch2R* takes advantage of the CZ CELLxGENE Discover API to get the download links of processed objects (*rds* and *h5ad* files) directly, the same as clicking the download button on the webpage. For processed objects in HCA, with links/filtered metadata and provided file extensions (e.g., *rds* and *h5ad*), *GEfetch2R* downloads the corresponding processed objects.

With processed objects in *rds* format, *GEfetch2R* loads them (*SeuratObjects*) to R, filters the *SeuratObjects* based on cell metadata and genes (CELLxGENE), and merges *SeuratObjects* if applicable. With processed objects in *RData* format, *GEfetch2R* dissects the elements within, extracts count matrices and metadata from standard (*SeuratObject, SingleCellExperiment, cell_data_set, CellDataSet, DESeqDataSet, DGEList*) or nonstandard objects, and loads them to R if applicable.

### Benchmark format conversion tools

To inspect the information kept after format conversion, we used the pbmc3k dataset analyzed by the standard *Seurat* and *Scanpy* workflows. The *SingleCellExperiment* used to inspect the information kept from *SingleCellExperiment* to *AnnData* was generated by *zellkonverter* (convert *AnnData* to *SingleCellExperiment*). The detailed code and output used to evaluate the retained information are provided in the Supplementary Note.

To examine the running time and scalability of format conversion tools, we used *GEfetch2R* to select and download 5 processed objects (both *rds* and *h5ad* files) with more than 1 million cells from CELLxGENE (Supplementary Table S1), then subsampled them to varied cell numbers: 1.2 million (1.2 M, 2 objects), 1 million (1 M), 800,000 (800 K), 600,000 (600 K), 500,000 (500 K), 300,000 (300 K), 200,000 (200 K), 100,000 (100 K), 80,000 (80 K), 60,000 (60 K), 50,000 (50 K), 30,000 (30 K), 20,000 (20 K), and 10,000 (10 K). Each format conversion tool was executed on all the 5 object sets (64 objects in total). For the same object set, we ensured consistency in the type of count matrices converted between different tools to avoid potential bias. All format conversion tools were executed on a server with 1 Intel Xeon Gold 5220R CPU, 512 GB RAM, and CentOS 7.5.1804 operating system. The running time (elapsed time) was recorded using the *system.time* command in R. To ensure the independence of each run, all commands were run in linear order on the Linux shell as R scripts.

### Format conversion between scRNA-seq objects

*GEfetch2R* uses Seurat to convert formats between SeuratObject and SingleCellExperiment, Seurat and SeuratDisk to convert formats between SeuratObject and loom, Seurat and SeuratWrappers to convert formats between SeuratObject and CellDataSet/cell_data_set, and LoomExperiment to convert formats between SingleCellExperiment and loom. For format conversion between AnnData and SeuratObject/SingleCellExperiment, all benchmarking tools are available in *GEfetch2R*.

## Results

### Overview of *GEfetch2R*

**Figure 1:**
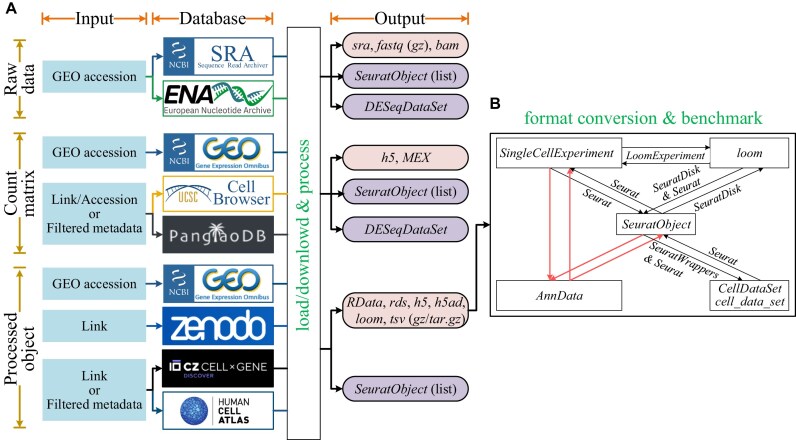
Overview of *GEfetch2R*. (A) Fetching and loading various data from public repositories to R. The download of raw data, count matrix, and processed object is indicated by ochre borders (horizontal). The input, supported databases, and final output are indicated by orange borders (vertical). The boxes in purple indicate R objects, and boxes in light pink indicate downloaded file formats. *MEX*: Market Exchange Format. (B) Format conversions supported by *GEfetch2R*. The boxes indicate scRNA-seq objects, the directional arrows between boxes indicate conversion streams between objects, the text above arrows represents the tool used for the conversion, and the red directional arrows without text indicate conversion streams that are benchmarked.

*GEfetch2R* supports downloading all 3 types of scRNA-seq data: raw data, count matrix, and processed object (Fig. 1). The databases corresponding to each data type, number of datasets/species/cells available, support formats, returned values, and other key features are summarized in Table 2. For fetching raw data, with a given GEO accession as input, *GEfetch2R* downloads *sra, fastq*, and *bam* files from SRA and ENA. In particular, the parameters for downloading 10x-related *fastq* and *bam* files have been optimized, such as downloading and formatting the *fastq* files to the *CellRanger* -required format and downloading *bam* files with original tags. With downloaded files, *GEfetch2R* processes them and loads the output to R (*SeuratObject* for 10x-generated data, *DESeqDataSet* for Smart-seq2 or bulk RNA-seq data). For fetching count matrices, with given accessions/links (GEO, UCSC Cell Browser, and PanglaoDB) or filtered metadata (UCSC Cell Browser and PanglaoDB) as input, *GEfetch2R* accesses count matrices and various annotations, loads them to R, and extracts a subset based on cell metadata and genes (UCSC Cell Browser and PanglaoDB). For fetching processed objects, with given accessions/links (GEO, Zenodo, CELLxGENE, and HCA) or filtered metadata (CELLxGENE and HCA) as input, *GEfetch2R* downloads processed objects in given file extensions, loads *rds* files (*SeuratObjects*) to R, extracts a subset based on cell metadata and genes (CELLxGENE), and dissects and extracts the *RData* files. The processed objects in other formats can be loaded to R/Python through format conversion.

**Table 2:** The databases supported corresponding to each data type.

RNA-seq type	Data type	Download mode	Database	Dataset	Species	Cell	Support format	Returned value (non-NULL)	Annotation	Subset	Parallel	Others
scRNA-seq	Raw data	Given accession	SRA, ENA	Unlimited	Unlimited	Unlimited	*fastq* (*gz*), *sra, bam*	*SeuratObject* (10x Genomics)/*DESeqDataSet* (Smart-seq2), failed sample metadata	Sample metadata	/	Yes	Multiple download and splitting methods, adapt to different scRNA-seq protocols, download status check, convert *bam* to *fastq*, merge multiple runs of a sample
	Count matrix	Given accession	GEO	Unlimited	Unlimited	Unlimited	*csv, tsv, txt, tab, xlsx, xls, h5, MEX* ^a^ (*gz*/*tar.gz*)	*SeuratObject*	Sample metadata	/	/	Generate count matrix from supplementary files, adapt to multiple feature counting tools
		Given accession, filtered metadata	PanglaoDB	1,368	Human/ mouse	5,586,348	/	*SeuratObject*	Sample and cell metadata	Cell type, gene	/	Summarize metadata, extract cell-type composition
		Given link, filtered metadata	UCSC Cell Browser	1,427	34	141,087,666	/	*SeuratObject*	Sample and cell metadata	Cell metadata, gene	/	Summarize metadata, extract cell-type composition and dimensionality reduction coordinates
	Processed object	Given accession	GEO	Unlimited	Unlimited	Unlimited	*RData, rds, h5ad, loom (gz/tar.gz)*	*SeuratObject*	Sample and cell metadata	/	/	Dissect and extract the *RData* files
		Given link	Zenodo	Unlimited	Unlimited	Unlimited	Unlimited	*SeuratObject*, failed doi information	Cell metadata	/	Yes	md5 check, dissect and extract the *RData* files
		Given link, filtered metadata	CELLxGENE	2,043	8	248,553,530	*rds*,^b^*h5ad*	*SeuratObject*, failed sample metadata	Sample and cell metadata	Cell metadata, gene	Yes	Download status check, summarize metadata, extract dimensionality reduction coordinates
		Given link, filtered metadata	Human Cell Atlas	546	4	~71,054,879	*RData, rds, h5, h5ad, loom, tsv* (*gz*/*tar.gz)*	*SeuratObject*, failed sample metadata	Sample and cell metadata	/	Yes	Download status check, summarize metadata, dissect and extract the *RData* files
Bulk RNA-seq	Raw data	Given accession	SRA, ENA	Unlimited	Unlimited	Unlimited	*fastq (gz), sra, bam*	*DESeqDataSet*, failed sample metadata	Sample metadata	/	Yes	Multiple download and splitting methods, download status check, convert *bam* to *fastq*, merge multiple runs of a sample
	Count matrix	Given accession	GEO	Unlimited	Unlimited	Unlimited	*csv, tsv, txt, tab, xlsx, xls* (*gz*/*tar.gz*)	*DESeqDataSet*	Sample metadata	/	/	Generate count matrix from supplementary files, adapt to multiple feature counting tools

a *MEX* stands for Market Exchange Format (barcodes.tsv.gz, features.tsv.gz/genes.tsv.gz, matrix.mtx.gz).

b CELLxGENE has deprecated *Seurat* downloads for versions after 2025 (earlier Seurat downloads remain available). *GEfetch2R* has stored *Seurat* download links in May 2024.

To enable the interoperability between scRNA-seq analysis tools and the integration of processed objects in diverse formats, *GEfetch2R* supports converting formats (i) between *SeuratObject* and *AnnData, SingleCellExperiment, CellDataSet*/*cell_data_set*, and *loom*; (ii) between *SingleCellExperiment* and *loom*; and (iii) between *SingleCellExperiment* and *AnnData*. Moreover, as several tools have been developed for the same conversion stream between *AnnData* and *SeuratObject/SingleCellExperiment*, we benchmarked their performance in terms of information kept, usability, running time, and scalability.

### Application of *GEfetch2R* in COVID-19 scRNA-seq atlas exploration

**Figure 2:**
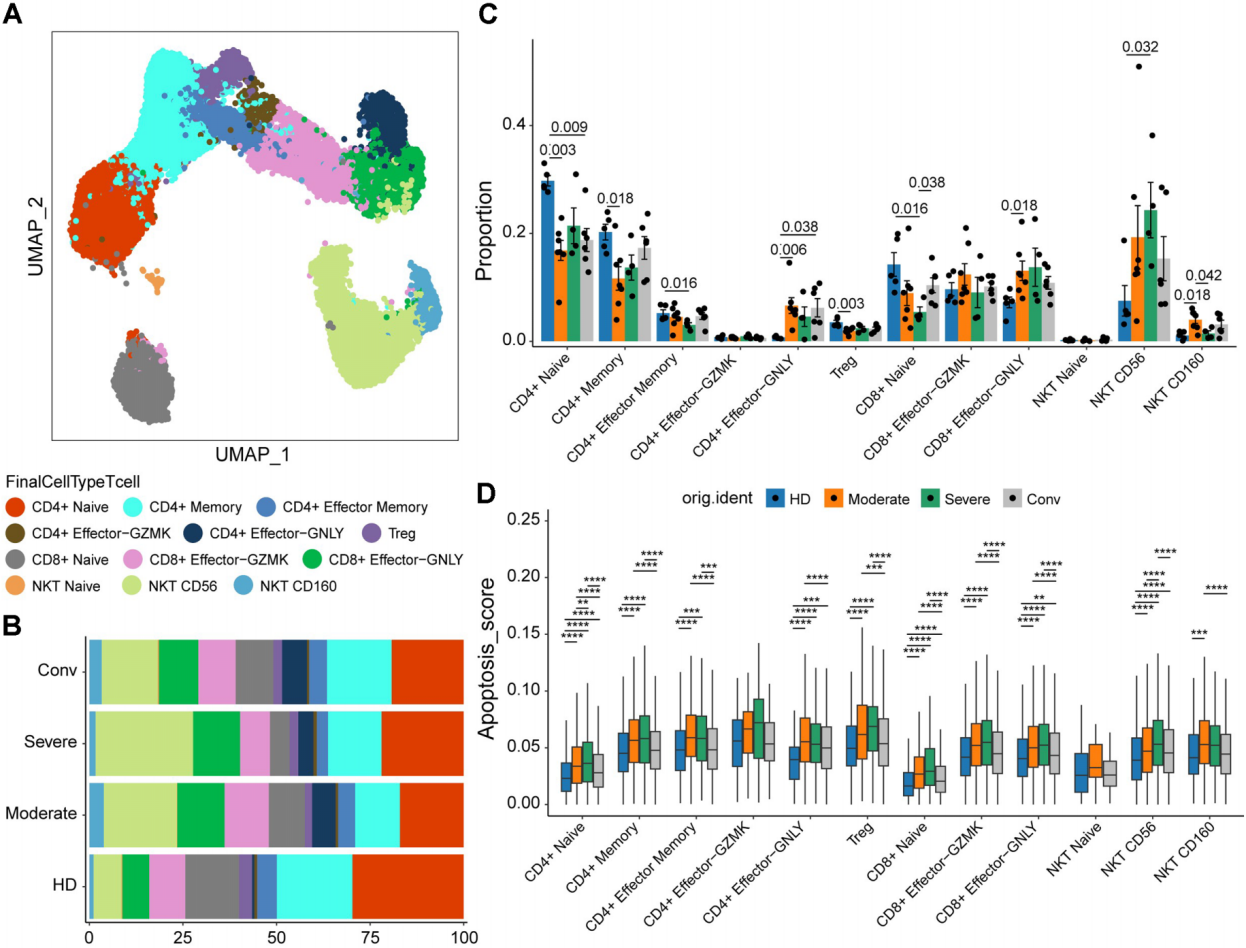
Application of *GEfetch2R* in COVID-19 scRNA-seq atlas exploration. (A) UMAP embedding of all T cells. Clusters in different colors represent T-cell subtypes. (B) The proportion of each cell type across HD, moderate, severe, and conv samples. (C) Differential cell-type composition analysis. All differences with *P* < 0.05 are labeled; a 2-sided unpaired Mann–Whitney *U* test was used for analysis. (D) The apoptosis scoring results. All differences with Bonferroni-adjusted *P* < 0.01 are indicated. **P < 0.01, ***P < 0.001, ^****^P < 0.0001, using a 2-sided unpaired Dunn’s test. NKT: natural killer T; Treg: regulatory T.

Coronavirus disease 2019 (COVID-19) is a highly contagious disease caused by severe acute respiratory syndrome coronavirus 2, with symptoms ranging from mild to severe [30]. Depicting the dynamic immune responses across symptom severities at the single-cell level is an effective method to understand COVID-19 progression. To demonstrate the utility of *GEfetch2R*, we applied it to download and explore all T cells of a COVID-19 scRNA-seq atlas [30] from the UCSC Cell Browser. As shown in Fig. 2A, there are 12 T-cell subtypes, including 6 subtypes of CD4^+^ T cells, 3 subtypes of CD8^+^ T cells, and 3 subtypes of natural killer T (NKT) cells. The cell-type composition analysis across 4 conditions (healthy donor [HD], moderate, severe, and convalescent [conv]) revealed that compared with HDs, the proportions of 4 CD4^+^ T subtypes (CD4^+^ naive, CD4^+^ memory, CD4^+^ effector memory, and regulatory T [Treg]) and the CD8^+^ naive subtype were significantly decreased in patients with COVID-19, and the proportion of the CD4*^+^* naive subtype was still significantly reduced in COVID-19 conv samples (Fig. 2B, C). Meanwhile, the proportions of CD4^+^ effector-GNLY, CD8^+^ effector-GNLY, NKT CD56, and NKT CD160 subtypes were significantly increased in patients with COVID-19, and the CD4^+^ effector-GNLY subtype was nearly absent in HDs but highly enriched in moderate, severe, and conv samples (Fig. 2B, C). The apoptosis scoring results showed that T cells in patients with COVID-19 generally had significantly increased apoptosis scores (Fig. 2D).

### Benchmark of format conversion tools

#### Information kept

**Figure 3:**
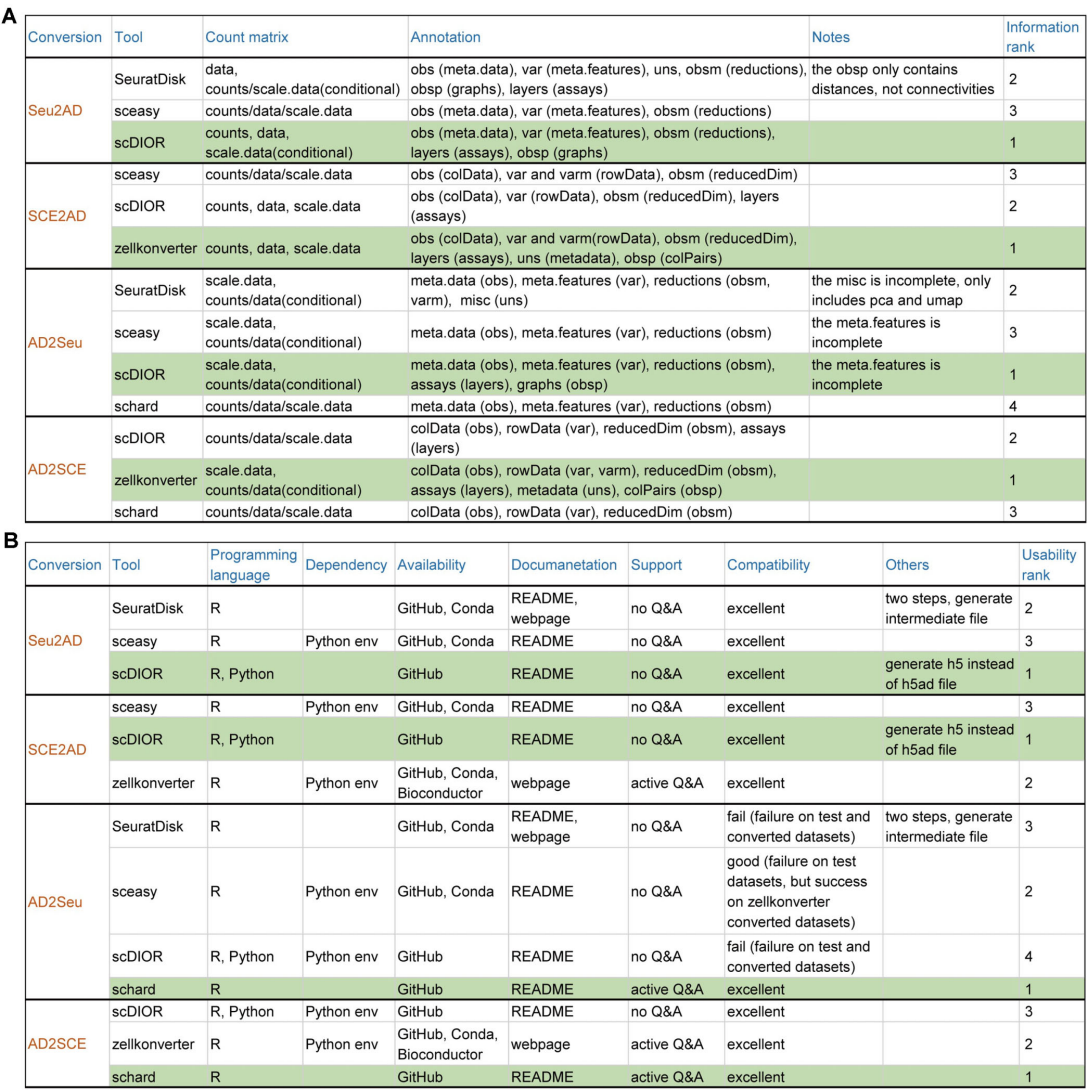
The information kept and usability of format conversion tools. (A) The information kept on format conversion tools in terms of count matrix and annotation. In the “Count matrix” column, “counts” represents the raw count matrix, “data” represents the normalized data matrix, and “scale.data” represents the scaled data matrix. In the “Annotation” column, the text outside/inside parentheses represents the data slot of the source/target object. (B) The usability of format conversion tools in terms of programming language, dependency, availability, documentation, support, and compatibility. Q&A: question and answer. The tool with the highest rank is marked in green.

We evaluated and ranked the information kept by format conversion tools in terms of the count matrix and annotation. Figure 3A and the Supplementary Note show our ranking reasoning and the overall information on the ranking of each tool. In Seu2AD, *scDIOR* can preserve all 3 count matrices and more annotations, thus ranking first. *SeuratDisk* can keep 2 of the 3 count matrices and more annotations, thus ranking second. In contrast, *sceasy* keeps only 1 count matrix and the fewest annotations, thus ranking last. In SCE2AD, *zellkonverter* and *scDIOR* both keep all 3 count matrices, while *zellkonverter* preserves the most comprehensive annotations, and thus *zellkonverter* ranks first. *sceasy* keeps only 1 count matrix and less comprehensive annotations, resulting in the lowest information kept rank. In AD2Seu, *scDIOR, sceasy*, and *SeuratDisk* can keep 2 of the 3 count matrices, which is more than *schard*. In addition, *scDIOR* preserves the most comprehensive annotations, thus ranking first. Both *schard* and *sceasy* retain the fewest annotations, and thus *schard* ranks last. In AD2SCE, *zellkonverter* can preserve 2 of the 3 count matrices and the most comprehensive annotations, thus ranking first. In contrast, both *schard* and *scDIOR* keep only 1 count matrix, and *schard* retains the fewest annotations, and thus *schard* ranks last.

#### Usability

We evaluated and ranked the usability of format conversion tools in terms of programming language, dependency, availability, documentation, support, and compatibility. Figure 3B shows our ranking rationale and the overall usability ranking of each tool. In Seu2AD, *sceasy* requires an additional Python environment, resulting in the lowest usability rank. *SeuratDisk* and *scDIOR* have comparable usability, but *scDIOR* can be used in both R and Python platforms, giving it a higher usability rank. In SCE2AD, *scDIOR* is the only tool that does not require an additional Python environment and can be used in both R and Python platforms, resulting in the highest usability rank. *zellkonverter* is hosted on Bioconductor and has active questions and answers (Q&As), thus ranking second. In AD2Seu, *schard* does not require an additional Python environment, has active Q&As, and is the only tool that runs successfully on all test data. Among the remaining 3 tools, *sceasy* runs successfully on *zellkonverter* converted *AnnData*, while the other tools still fail. Based on the above results, *schard* ranks first and *sceasy* ranks second in usability, and the subsequent running time is recorded on *zellkonverter* converted *AnnData*. In AD2SCE, *schard* has active Q&As and is the only tool that does not require an additional Python environment, resulting in the highest usability rank. *zellkonverter* and *scDIOR* have comparable usability, but the former is slightly better. In detail, *zellkonverter* is hosted on Bioconductor and has active Q&As, while *scDIOR* can be used in both R and Python platforms.

#### Running time and scalability

**Figure 4:**
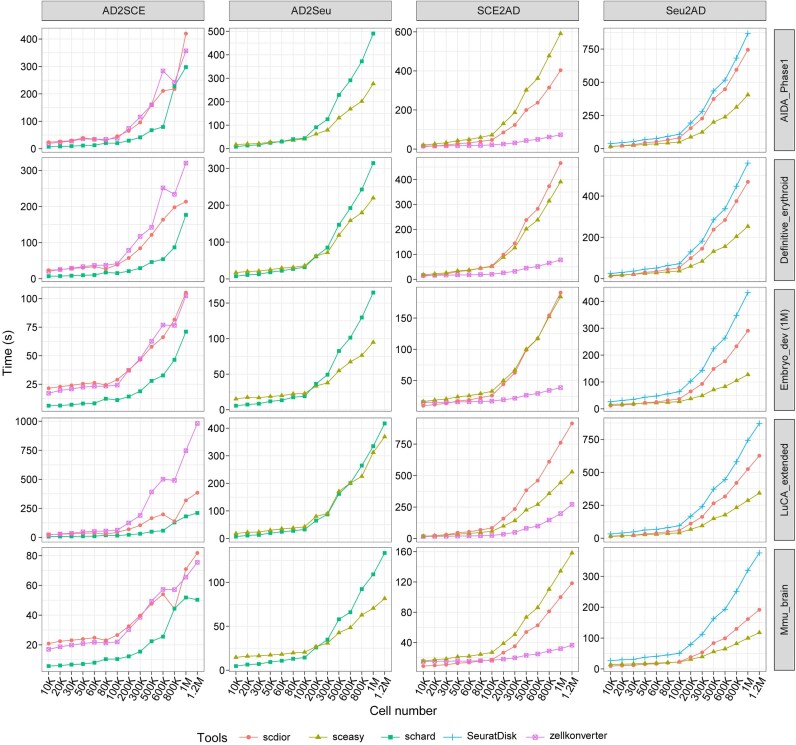
The running time of format conversion tools. There are 20 subplots, distributed in 4 rows and 5 columns. Each row represents an object set. Each column represents the same conversion stream. The x-axis represents the cell number (K: thousand, M: million), and the y-axis indicates the running time.

In Supplementary Table S2, we summarized the running time of the format conversion tools for converting between *AnnData* and *SeuratObject*/*SingleCellExperiment* on 5 object sets (64 objects in total). Figure 4 shows that in AD2SCE, *schard* is always the fastest tool across all 5 object sets, while *zellkonverter* is the slowest tool in 2 of 5 object sets. In AD2Seu, when the cell number is smaller than 200 K, *schard* is faster than *sceasy* or comparable in speed, but *sceasy* is significantly faster than *schard* when the cell number exceeds 200 K. This alternation means that *sceasy* has better scalability than *schard*. In SCE2AD, *zellkonverter* is always the fastest tool across all 5 object sets, while *sceasy* and *scDIOR* are the slowest tools in 2 of 5 object sets. In Seu2AD, across all 5 object sets, *sceasy* is always the fastest tool, while *SeuratDisk* is always the slowest tool.

#### Comparison of *GEfetch2R* with other similar tools

Table 1 shows a feature comparison of *GEfetch2R* with other tools that are capable of downloading data from public repositories. *GEfetch2R* distinguishes itself in the following aspects. First, *GEfetch2R* supports downloading the most diverse data types (raw data, count matrix, and processed object), and each data type can be downloaded from multiple public repositories. This benefits users by enabling them to collect data more comprehensively and choose appropriate data types for different needs. For quick data exploration, downloading processed objects is a good choice, whereas for fine data integration to draw conclusions, downloading raw data or count matrices can help reduce the bias. Second, *GEfetch2R* adopts multiple methods to speed up downloading, including Aspera support, parallel downloading *fastq* files, and parallel splitting *sra* files into *fastq* files. Third, *GEfetch2R* supports downloading data of both scRNA-seq and bulk RNA-seq and considers the characteristics of data generated by different protocols. For 10x-generated data, *GEfetch2R* downloads and formats the *fastq* files to the *CellRanger*-required format, downloads *bam* files with original tags, converts *bam* files to *fastq* files using *bamtofastq*, and aligns *fastq* files to the reference genome using *CellRanger*. For Smart-seq2 and bulk RNA-seq data, *GEfetch2R* converts *bam* files to *fastq* files using *samtools* and aligns *fastq* files to the reference genome using *STAR*. This expands the scope of use of *GEfetch2R* and makes the data it downloads easier to use. Fourth, besides data download, *GEfetch2R* can process the downloaded data, load the count matrices/annotations/*rds* files to R (*SeuratObject*/*DESeqDataSet*), extract a subset of the *SeuratObject* based on cell metadata and genes, and dissect and extract the *RData* files. This greatly distinguishes *GEfetch2R* from other tools, which are pure data downloaders. Lastly, *GEfetch2R* provides the most comprehensive object format conversions and benchmarks the format conversion tools for converting between *AnnData* and *SeuratObject*/*SingleCellExperiment*. This enables users to integrate multiple objects in diverse formats, bridges the widely used scRNA-seq analysis tools, and guides the selection of format conversion tools.

## Conclusions and Discussion

*GEfetch2R* is an R package that facilitates researchers in accessing and exploring existing gene expression data from various public repositories. As a data downloader, *GEfetch2R* supports downloading the most diverse scRNA-seq data types, including raw data (SRA and ENA), count matrix (GEO, UCSC Cell Browser, and PanglaoDB), and processed objects (GEO, Zenodo, CELLxGENE, and HCA). Besides the data download ability, *GEfetch2R* can process the downloaded data, load the count matrices/annotations/*rds* files to R (*SeuratObject*/*DESeqDataSet*), extract a subset of the *SeuratObject* based on cell metadata and genes, and dissect and extract the *RData* files. Furthermore, to enable the integration of scRNA-seq data and the interoperability among different analysis tools, *GEfetch2R* provides the most comprehensive format conversions across different scRNA-seq objects, including *SeuratObject, AnnData, SingleCellExperiment, CellDataSet*/*cell_data_set*, and *loom*. In particular, *GEfetch2R* benchmarks the format conversion tools for converting between *SeuratObject*/*SingleCellExperiment* and *AnnData*. In Seu2AD, *scDIOR* has the best performance in terms of information kept and usability, and *sceasy* is always the fastest tool. In SCE2AD, *zellkonverter* has the highest information kept rank, *scDIOR* is the best in usability, and *zellkonverter* is consistently the fastest tool. In AD2Seu, *scDIOR* has the best performance in terms of information kept, *schard* is the best in usability, and *sceasy* has better scalability than *schard*. In AD2SCE, *zellkonverter* has the highest information kept rank, *schard* is the best in terms of usability and speed.

In addition to fetching scRNA-seq data, *GEfetch2R* can also be used to download raw data (SRA and ENA) and count matrices (GEO) of bulk RNA-seq, process the downloaded data, and load output/downloaded count matrices to R (*DESeqDataSet*).

Currently, several aspects of *GEfetch2R* can be improved. First, *GEfetch2R* only supports processing the Smart-seq2 and 10x-generated raw data, and users can download the raw data generated by other scRNA-seq protocols using *GEfetch2R*; however, the subsequent process is not available. Second, many widely used scRNA-seq databases are not supported by *GEfetch2R*, such as Single Cell Portal [31] and gEAR [32]. We will actively develop to support more scRNA-seq protocols and databases. Besides, since the APIs of supported databases may change, we will update *GEfetch2R* in a timely manner to ensure continued use.

## Availability and Requirements

- Project name: *GEfetch2R*
- Project homepage: https://github.com/showteeth/GEfetch2R
- Software documentation: https://showteeth.github.io/GEfetch2R
- Docker image: https://hub.docker.com/r/soyabean/gefetch2r
- Operating system(s): Platform independent
- Programming language: R
- Other requirements: R 2.10 or higher, Python 3.7.3 or higher (format conversion)
- License: GPL-3.0
- RRID:SCR_026714
- biotoolsID: gefetch2r

## Supplementary Material

giag039_Supplemental_Files

giag039_Authors_Response_To_Reviewer_Comments_original_submission

giag039_GIGA-D-25-00313_original_submission

giag039_GIGA-D-25-00313_Revision_1

giag039_Reviewer_1_Report_original_submission Reviewer 1 -- 9/29/2025

giag039_Reviewer_1_Report_revision_1 Reviewer 1 -- 3/16/2026

giag039_Reviewer_2_Report_original_submission Reviewer 2 -- 12/3/2025

giag039_Reviewer_2_Report_revision_1 Reviewer 2 -- 2/27/2026

## Data Availability

The datasets used in this study are freely available from UCSC Cell Browser (“COVID-19 Immunological Response”/“T-cells”), *SeuratData* (built-in dataset: pbmc3k.final) [33], 10x Genomics (3k PBMCs from a healthy donor), and CELLxGENE (Supplementary Table S1). All scripts used for the COVID-19 scRNA-seq atlas exploration and benchmark of format conversion tools, including downloading, processing, subsampling, running, and visualizing, are available in the GitHub repository [34]. Snapshots of the code and data are available in Software Heritage [35]. All supplementary material is available in the *GigaScience* repository, GigaDB [36].

## Additional Files

**Supplementary Note**. Step-by-step code and output showing information kept by format conversion tools.

**Supplementary Fig. S1**. The detailed workflow of *GEfetch2R*.

**Supplementary Table S1**. The datasets used for benchmarking format conversion tools.

**Supplementary Table S2**. The running time of the format conversion tools on 5 object sets.

## Abbreviations

10x: 10x Genomics; AD2SCE: from AnnData to SingleCellExperiment; AD2Seu: from AnnData to SeuratObject; conv: convalescent; COVID-19: coronavirus disease 2019; ENA: European Nucleotide Archive; GEO: Gene Expression Omnibus; HCA: Human Cell Atlas; HD: healthy donor; K: thousand; M: million; NKT: natural killer T; Q&As: questions and answers; SCE2AD: from SingleCellExperiment to AnnData; scRNA-seq: single-cell RNA sequencing; Seu2AD: from SeuratObject to AnnData; SRA: Sequence Read Archive; Treg: regulatory T.
