# Supplementary material for: GEfetch2R: fetching single-cell/bulk RNA-seq data from public repositories to R and benchmarking the subsequent format conversion tools
Source: Gigascience. 2026 Apr 8;15:giag039. doi: 10.1093/gigascience/giag039 (PMC13147242; doi:10.1093/gigascience/giag039)
Supplement: giag039_GIGA-D-25-00313_Revision_1 [file giag039_giga-d-25-00313_revision_1.pdf]

GigaScience

GEfetch2R: fetching single-cell/bulk RNA-seq data from public repositories to R and benchmarking the subsequent format conversion tools

--Manuscript Draft--

Manuscript Number:	GIGA-D-25-00313R1
Full Title:	GEfetch2R: fetching single-cell/bulk RNA-seq data from public repositories to R and benchmarking the subsequent format conversion tools
Article Type:	Technical Note
Funding Information:	Institute of Microbiology, Chinese Academy of Sciences (the start-up funds) — Prof. Jiaxin Gao<br>National Natural Science Foundation of China (22050004) — Prof. Jianbin Wang<br>Chinese Academy of Sciences Project for Young Scientists in Basic Research (No. YSBR-111) — Prof. Jiaxin Gao
Abstract:	Background<br><br>Downloading and reanalyzing the existing single-cell RNA sequencing (scRNA-seq) data provides an efficient choice to gain clues and new insights. However, no tool can fetch the diverse scRNA-seq data types (raw data, count matrix, and processed object) distributed in various repositories, process and load the downloaded data to R, convert formats between scRNA-seq objects, and benchmark the format conversion tools.<br><br>Findings<br><br>Here, we present GEfetch2R, an R package with Docker image to (i) download diverse scRNA-seq data types, including raw data (SRA and ENA), count matrix (GEO, UCSC Cell Browser, and PanglaoDB), and processed object (GEO, Zenodo, CELLxGENE, and HCA); (ii) process the downloaded data, load the count matrices, annotations, and rds files to R (SeuratObject/DESeqDataSet), filter the SeuratObject based on cell metadata and genes, and dissect and extract the RData files; (iii) convert formats between the widely used scRNA-seq objects, including SeuratObject, AnnData, SingleCellExperiment, CellDataSet/cell_data_set, and loom, and benchmark format conversion tools in terms of information kept, usability, running time, and scalability to guide the tool selection. Furthermore, GEfetch2R can also download, process, and load bulk RNA-seq raw data (SRA and ENA) and count matrices (GEO) to R (DESeqDataSet).<br><br>Conclusions<br><br>GEfetch2R is an R package dedicated to facilitating researchers to access and explore the existing gene expression data from various public repositories. It can function as a data downloader (supports all three scRNA-seq and two bulk RNA-seq data types), a data processor (processes and loads the output/downloaded count matrices and annotations to R), and an object format converter (between the widely used scRNA-seq objects).
Corresponding Author:	Jiaxin Gao<br>Chinese Academy of Sciences<br>Beijing, CHINA
Corresponding Author Secondary Information:	
Corresponding Author's Institution:	Chinese Academy of Sciences
Corresponding Author's Secondary Institution:	
First Author:	Yabing Song

Powered by Editorial Manager® and ProduXion Manager® from Aries Systems Corporation

**First Author Secondary Information:**	
**Order of Authors:**	Yabing Song
	Jianbin Wang
	Jiaxin Gao
**Order of Authors Secondary Information:**	
**Response to Reviewers:**	Replies to reviewers' comments (PDF version and Supplementary Data/Table can be accessed via https://github.com/showteeth/GEfetch2R/raw/refs/heads/main/man/benchmark/Response%20to%20Reviewers.zip):<br><br>>We thank all reviewers for taking the time to read our manuscript and for giving suggestions to improve it. We truly appreciate the positive assessment of our work. In this document, we attempt to address all concerns and questions raised by reviewers.<br><br>--<br><br>Reviewer #1: The manuscript presents GEfetch2R, an R package (with a Docker image) that fetches scRNA-seq and bulk RNA-seq data from multiple repositories, loads the data into R objects, and benchmarks format-conversion tools. The problem addressed is real and important; the implementation appears practical and well documented. I see strong potential for adoption.<br><br>>We are deeply grateful for your concise summary and your profound interest in our study.<br><br>Major comments<br>1. Robust cross-repository support for .RData files<br>While GEfetch2R lists rdata among supported extensions for Zenodo and HCA, many GEO submissions and other archives still provide processed data exclusively as .RData, often bundling matrices and metadata in heterogeneous objects. Please add an explicit, repository-agnostic .RData ingestion path with: (i) automatic object introspection, (ii) standardized extraction of matrices/metadata, (iii) graceful fallbacks with clear diagnostics for non-standard objects, and (iv) reproducible examples. This materially increases real-world coverage.<br><br>>In response to the reviewer's suggestion, we have made the following improvements: 1) added the function ParseGEOProcessed to parse the processed objects (rds (load into R automatically if it's SeuratObject), RData, loom, and h5ad) from GEO; 2) added the function LoadRData to dissect and extract the RData files. The LoadRData loads the RData file to a separate environment, distinguishes the class of each object available, and processes the objects according to the following logic (the detailed procedures are attached as Supplementary Data 1 (https://github.com/showteeth/GEfetch2R/raw/refs/heads/main/man/benchmark/Response%20to%20Reviewers.zip) and can be accessed via https://showteeth.github.io/GEfetch2R/articles/DownloadObjects.html#process-rdata-files):<br>*if widely used scRNA-seq objects (SeuratObject (Seurat package), seuratobject (Seurat v2 package), SingleCellExperiment (SingleCellExperiment package), cell_data_set (Monocle v3 package), CellDataSet (Monocle package)) or bulk RNA-seq objects (DESeqDataSet (DESeq2 package), DGEList (edgeR package)) exist, LoadRData automatically distinguishes the object class and extracts the raw count matrix, normalized count matrix, scaled count matrix, and metadata.<br>*else if non-standard objects (matrix, data.frame, dgCMatrix, dgRMatrix, dgTMatrix) exist, for matrix/data.frame objects, check the class of each column value and the name of every column, treat objects with numeric/integer values as count matrices, treat objects with sample group/annotation related column names as sample metadata, otherwise display the structure of the objects and load the objects to R for manually check; for dgCMatrix/dgRMatrix/dgTMatrix objects, treat them as count matrices directly.<br>*else print the first six (or less) elements of the objects.<br>>The cross-repository and reproducible examples are attached as Supplementary Data

Powered by Editorial Manager® and ProduXion Manager® from Aries Systems Corporation

1
(https://github.com/showteeth/GEfetch2R/raw/refs/heads/main/man/benchmark/Response%20to%20Reviewers.zip) and can be accessed via https://showteeth.github.io/GEfetch2R/articles/DownloadObjects.html#process-rdata-files. For more examples and evaluation results, please refer to the next response.

2. Large-scale, automated evaluation on ~100 scRNA-seq datasets
Beyond the single COVID-19 application and the conversion benchmark, please include a systematic "fetch success-rate" study across ~100 GEO scRNA-seq datasets. Provide a Dockerized workflow (publicly available) that periodically attempts end-to-end retrieval (raw / count / processed) and reports success/failure rates stratified by repository and file type, with resource/time footprints and categorized failure causes. Given heterogeneous deposition practices, even ~50% overall success would be informative.

>We agree that large-scale evaluation is crucial for testing the usability of GEfetch2R. However, a GEO accession could contain several supplementary files, which may include files unrelated to the count matrix. GEfetch2R usually requires users to specify a supplementary file (regardless of format, whether it's compressed or archived) as the file storing the count matrix. These bottlenecks make automated evaluation difficult. And as the second reviewer mentioned, users typically need GEfetch2R to access data for a specified accession. Thus, we conducted the large-scale evaluation by manually searching and selecting GEO accessions. It should be noted that this selection was performed before running GEfetch2R. The number of evaluation accessions selected for each category depends on the complexity of the supplementary files.
>The evaluation results, separated into "download" and "create R object", are attached as Table R1, Supplementary Data 2 and 3 (https://github.com/showteeth/GEfetch2R/raw/refs/heads/main/man/benchmark/Response%20to%20Reviewers.zip). In summary, for Smart-seq2 scRNA-seq, GEfetch2R supports loading count matrices from files in diverse formats (csv/tsv/txt/tab/xlsx/xls, whether compressed or/and archived, whether single file or multiple files) and files generated by various feature counting tools (STAR, htseq-count, featureCounts), removing preset or user-specified useless columns, automatically transposing the transposed count matrix (the number of rows/genes is less than the number of columns/samples), and creating the SeuratObject. The failure causes can be categorized as "file with comment lines", "first column isn't genes/features", and "unspecified numeric column". The success rates are 93.9% and 100%. For 10x Genomics scRNA-seq, GEfetch2R can create SeuratObjects from count matrices stored in h5 files (whether compressed or/and archived) and files in MEX format (whether compressed or/and archived). GEfetch2R also supports count matrices generated by scRNA-seq platforms other than 10x Genomics, which have a similar output structure (MEX format) to 10x Genomics, e.g., SeekOne and MobiDrop. The failure causes can be categorized as "incorrect file name", "missing file", "unsupported format", and "parameter adjustment". The success rates are 94.0% and 98.4%. GEfetch2R supports downloading processed objects in RData, rds, h5ad, and loom formats (whether compressed or/and archived), loading SeuratObjects stored in rds files to R, and dissecting and extracting the RData files. The success rates are 100% and 92.9%-100%. The failure cause is "key word misidentification" and the Seurat version is a risk factor. The overall success rates are 96.2% and 98.6%. The evaluation codes (GEO_evaluation_download.R, GEO_evaluation_createObject.R) are in the Docker image (soyabean/gefetch2r:1.2, path: /home/rstudio/GEOBench) and the GEfetch2R GitHub repository (https://github.com/showteeth/GEfetch2R/tree/main/man/benchmark).
>To further facilitate users in checking the usability of GEfetch2R, we have provided the function CheckAPI to automatically check the API availability of various databases (https://showteeth.github.io/GEfetch2R/articles/DownloadRaw.html#check-api, https://showteeth.github.io/GEfetch2R/articles/DownloadMatrices.html#check-api, https://showteeth.github.io/GEfetch2R/articles/DownloadObjects.html#check-api).

3. Another very important point is to provide a Dockerfile together with the Docker.

>Thanks for your reminder. We now provide the Dockerfile and necessary files (e.g. Docker_test.R: codes for testing the usability of the image; GEO_evaluation_download.R and GEO_evaluation_createObject.R: codes for

conducting the large-scale evaluation) via GitHub (https://github.com/showteeth/GEfetch2R/tree/main/Docker/).

Minor revisions
"altas" → atlas (COVID-19 section title/caption).
"Count maatrix" → Count matrix (Figure 3 caption/table column).
"PanglanDB" → PanglaoDB (tables).
Consistency: keep SeuratObject (not "Seurat object"); keep rds lowercase;

>We apologize for the previous misspelling and inconsistency. All the above mistakes have been corrected in the revised manuscript (lines 270, 276, 291, 466; line 320; Table 1, Table 2, documentation, website; documentation, website). We also further checked the manuscript to avoid similar issues.

Reviewer #2: Every day, more than 30 single cell datasets are published. Almost none of them in a homogenous format, and not available anywhere but in obscure archives. Making the data more accessible is a major problem, which NIH and NBCI in particular bungled. They could have funded one group to make this easier or tell GEO to do it, but they didn't. So now we're getting packages like this one here who spend years on fixing this problem with complex commands.
So the authors here provide yet another single cell downloader/processing package, but this one compares itself to the previous ones, and includes steps for aligning and for making the expression matrix. This type of software is really needed, with more than a dozen different single cell websites, it's annoying to convert data all the time.

>Many thanks for your precise summary of the current data acquisition status and your recognition in our study.

Major:
My main feedback is that the documentation is somewhat hard to find and that there is no "quick start" tutorial. The main question that the user who comes to your website has is "how do I import dataset X now" ? For that you need a docs page linked from the top of your Github where it walks the user through importing a dataset. Not finding it, because we can use Google for that. Once i have a link to a GEO dataset, or a cell browser dataset, how can I load the expression matrix? I think that should be covered. I needed to click around quite a bit to find this page: https://showteeth.github.io/GEfetch2R/articles/DownloadMatrices.html but it confuses the user by telling me how to download all datasets and finding the right one, but in most cases, I already have the right one. And this page should be easier to find.

>This is an excellent point. As suggested, we have added the "Quick start" tutorial in the GitHub README.md (https://github.com/showteeth/GEfetch2R?tab=readme-ov-file#quick-start-1) and in a new webpage of the GEfetch2R website (https://showteeth.github.io/GEfetch2R/articles/QuickStart.html). Besides, we have also added prominent links for users to conveniently access this tutorial at the very top of the GitHub README.md (https://github.com/showteeth/GEfetch2R?tab=readme-ov-file#quick-start) and the welcome page of the GEfetch2R website (https://showteeth.github.io/GEfetch2R/index.html).
>In the "Quick start" tutorial, we provide commands to access data based on the categorization of databases instead of data types, which makes it easier for users to retrieve data based on the database-specific links or accessions. For databases where the metadata of all datasets or projects are available (PanglaoDB, UCSC Cell Browser, CELLxGENE, and Human Cell Atlas), we classify data access strategies into two types: based on given accessions/links (e.g. https://showteeth.github.io/GEfetch2R/articles/QuickStart.html#given-dataset-1) and based on filtered metadata (e.g. https://showteeth.github.io/GEfetch2R/articles/QuickStart.html#filter-samples-based-on-metadata-1). For databases such as GEO and Zenodo, we provide the data access strategy based on given accessions or links (e.g. https://showteeth.github.io/GEfetch2R/articles/QuickStart.html#zenodo-processed-object). We hope that those strategies can simultaneously meet users' needs for downloading specified data and downloading data with similar characteristics in bulk.

Powered by Editorial Manager® and ProduXion Manager® from Aries Systems Corporation

Minor:
1. One database that you are missing entirely is Nemo analytics alias gEar, but that's totally OK. The manuscript is very complete and covers the main features and justifies its use of other packages, which in itself is already interesting.

>Thank you again for your supportive appraisal. The gEAR portal (https://umgear.org/dataset_explorer.html) is an amazing platform for data deposition, display, analysis and interrogation, with a focus on allowing users to customize the site and their own data displays. I have figured out the way to download the processed objects by providing shareable links. However, after reading the article and conducting a cursory search of issues on GitHub, I couldn't find an available API. I'm not sure if it's allowed to directly provide a download function (I have created an issue on GitHub: https://github.com/IGS/gEAR/issues/1176). If it is, we would be happy to offer such a function in the next release. In the revised manuscript (lines 420-421), I regard it as an important aspect for future improvement.

2. This page https://showteeth.github.io/GEfetch2R/articles/DownloadMatrices.html does not cover Cellxgene. I don't know how that's possible: you added Cellxgene support, but then forgot to document it?

>The CELLxGENE database provides processed objects (h5ad and rds) for downloading, so the tutorial can be found at https://showteeth.github.io/GEfetch2R/articles/DownloadObjects.html. To avoid possible confusion, we now list the corresponding databases alongside the tutorials (https://github.com/showteeth/GEfetch2R?tab=readme-ov-file#vignette, https://showteeth.github.io/GEfetch2R/index.html#vignette).

3. The https://showteeth.github.io/GEfetch2R/articles/DownloadRaw.html, just like the other page above, requires a ton of settings. It would be nice if this was simpler. The docker container could set environment variables so I don't have to provide the directories for the SRA tools all the time. Right now, the package is far from easy to run. I understand that this is a difficult task, but some automated assistant, wrapper function, where I can just run downloadFileAndAlign(accession, genome) would be really nice. The website can be guessed from the accession and the 10x or not format can be guessed from the metadata.

>Following the reviewer's suggestion, we have added a wrapper function DownloadFastq2R to simplify the procedures from downloading data to loading into R, while minimizing the number of parameters. The DownloadFastq2R can extract all runs under a given accession, automatically identify the RNA-seq type (10x Genomics scRNA-seq, bulk RNA-seq, Smart-seq2 scRNA-seq/mini-bulk RNA-seq) of each run, download fastq files from ENA, perform read alignment and feature counting (merge multiple runs of a sample), and load the results to R (SeuratObject/DESeqDataSet).
The DownloadFastq2R automatically identify the RNA-seq type according to the following logic:
*filter the library_strategy field ("rna-seq", "scrna-seq", "snrna-seq") to remove non-RNA-seq runs
*if library_source field is "transcriptomic single cell" or library_strategy field is in c("scrna-seq", "snrna-seq") or key words ("scRNA", "snRNA", "single.cell", "single.nuclei", "single.nucleus", "singlecell", "singlenuclei", "singlenucleus", "10X.Genomics", "10XGenomics", "Smart.seq2", "Smartseq2") appear in title|source_name|characteristics|description fields, the run is classified as scRNA-seq:
-if key words ("10X.Genomics", "Cell.Ranger", "10XGenomics", "CellRanger") appear in any field, the run is classified as 10x Genomics scRNA-seq
-if key words ("Smart.seq2", "Smartseq2") appear in any field, the run is classified as Smart-seq2 scRNA-seq/mini bulk RNA-seq
-else the run will skip subsequent processing steps
*else the run is classified as bulk RNA-seq
>If the above automatic identification failed, users can specify the RNA-seq type via parameter "force.type".
>The key parameters needed are GEO accession ("acce")/GSM accessions ("gsm"), path to the mapper ("star.path"/"cellranger.path", can be automatically detected), and path to the reference genome ("star.ref"/"cellranger.ref"). The examples can be found

Powered by Editorial Manager® and ProduXion Manager® from Aries Systems Corporation

	at https://showteeth.github.io/GEfetch2R/articles/DownloadRaw.html#one-step-wrapper.<br>>Additionally, we are committed to simplifying the data access procedures from other databases. Currently, users only need to provide links/accessions to retrieve count matrices or processed objects from PanglaoDB, UCSC Cell Browser, Zenodo, CELLxGENE, Human Cell Atlas.<br><br>4. With packages like this, more than the initial release, what's most important to get users is support and updates. I hope that even if the author is a grad student, that the lab's PI can continue supporting this package. Otherwise this risks just becoming another abandoned package.<br><br>>We agree that support and updates are crucial for the usability of GEfetch2R. The biggest factor affecting the functionality of GEfetch2R is the availability of the APIs. We briefly reviewed the GitHub commit records (the initial commit was on Jul 12, 2023), the APIs to access GEO, PanglaoDB, and UCSC Cell Browser are quite robust with no failures, and the APIs to access Zenodo, CELLxGENE, and Human Cell Atlas had minor changes in the past. To conveniently and quickly check the availability of APIs, we have added the function CheckAPI (https://showteeth.github.io/GEfetch2R/articles/DownloadRaw.html#check-api, https://showteeth.github.io/GEfetch2R/articles/DownloadMatrices.html#check-api, https://showteeth.github.io/GEfetch2R/articles/DownloadObjects.html#check-api).<br>>As a frequently used tool in our laboratory, we will ensure its ongoing support and timely updates in the future, as long as the continuous availability of database APIs. We believe that the publication of this tool will further ignite our enthusiasm and attract more users to provide feedback and suggestions for improvement, thereby refining the tool.
**Additional Information:**	
**Question**	**Response**
Are you submitting this manuscript to a special series or article collection?	No
**Experimental design and statistics**<br><br>Full details of the experimental design and statistical methods used should be given in the Methods section, as detailed in our Minimum Standards Reporting Checklist. Information essential to interpreting the data presented should be made available in the figure legends.<br><br>Have you included all the information requested in your manuscript?	Yes
**Resources**<br><br>A description of all resources used, including antibodies, cell lines, animals and software tools, with enough information to allow them to be uniquely identified, should be included in the Methods section. Authors are strongly	Yes

Powered by Editorial Manager® and ProduXion Manager® from Aries Systems Corporation

encouraged to cite Research Resource Identifiers (RRIDs) for antibodies, model organisms and tools, where possible.<br><br>Have you included the information requested as detailed in our Minimum Standards Reporting Checklist?	
**Availability of data and materials**<br><br>All datasets and code on which the conclusions of the paper rely must be either included in your submission or deposited in publicly available repositories (where available and ethically appropriate), referencing such data using a unique identifier in the references and in the “Availability of Data and Materials” section of your manuscript.<br><br>Have you have met the above requirement as detailed in our Minimum Standards Reporting Checklist?	Yes
GigaScience has policies and guidelines in place for the use of generative AI-writing tools such as ChatGPT. If you have used such writing tools to assist with writing the manuscript this must be declared and cited in the text. Authors should not list AI-writing tools and other AI-assisted technologies as an author or co-author and should acknowledge that they are fully responsible for text generated or refined by AI-writing tools.<p><br><br>A summary of use (particularly in the introduction or among methods) needs to be included at the end of the paper, and the outputs should also be included as a supplementary file hosted in GigaDB or other open repositories. Please read our guidelines for more information. <p><br><br>By submitting to GigaScience, you are aware of the journal's AI-writing tools policy, and if you have declared use of such tools below, you have acknowledged this where appropriate in your manuscript and have made a summary of use and	No

Powered by Editorial Manager® and ProduXion Manager® from Aries Systems Corporation

outputs available. <p><br><b>AI-assisted writing tools have been used in the preparation of this manuscript?	

Manuscript
Click here to access/download;Manuscript;GEfetch2R_manuscript_rev.docx

GEfetch2R: fetching single-cell/bulk RNA-seq data from public repositories to R and benchmarking the subsequent format conversion tools

Yabing Song[1], Jianbin Wang[2*], Jiaxin Gao[1*]

[1]State Key Laboratory of Microbial Diversity and Innovative Utilization, Institute of Microbiology, Chinese Academy of Sciences, Beijing, China

[2]School of Life Sciences, Tsinghua University, Beijing, China.

*Address correspondence to Jianbin Wang, jianbinwang@tsinghua.edu.cn and Jiaxin Gao, gaojx@im.ac.cn

Key words: single-cell/bulk RNA-seq, data download, format conversion, tool benchmark, software

Yabing Song[0000-0003-1352-2994]; Jianbin Wang[0000-0001-6725-7925]; Jiaxin Gao[0000-0001-6161-4629]

Abstract

Background

Downloading and reanalyzing the existing single-cell RNA sequencing (scRNA-seq) data provides an efficient choice to gain clues and new insights. However, no tool can fetch the diverse scRNA-seq data types (raw data, count matrix, and processed object) distributed in various repositories, process and load the downloaded data to R, convert formats between scRNA-seq objects, and benchmark the format conversion tools.

Findings

Here, we present *GEfetch2R*, an R package with Docker image to (i) download diverse scRNA-seq data types, including raw data (SRA and ENA), count matrix (GEO, UCSC Cell Browser, and PanglaoDB), and processed object (GEO, Zenodo, CELLxGENE, and HCA); (ii) process the downloaded data, load the count matrices, annotations, and *rds* files to R (*SeuratObject*/*DESeqDataSet*), filter the *SeuratObject* based on cell metadata and genes, and dissect and extract the *RData* files; (iii) convert formats between the widely used scRNA-seq objects, including *SeuratObject*, *AnnData*, *SingleCellExperiment*, *CellDataSet*/*cell_data_set*, and *loom*, and benchmark format conversion tools in terms of information kept, usability, running time, and scalability to guide the tool selection. Furthermore, *GEfetch2R* can also download, process, and load bulk RNA-seq raw data (SRA and ENA) and count matrices (GEO) to R (*DESeqDataSet*).

Conclusions

GEfetch2R is an R package dedicated to facilitating researchers to access and explore the existing gene expression data from various public repositories. It can function as a data downloader (supports all three scRNA-seq and two bulk RNA-seq data types), a data processor (processes and loads the output/downloaded count matrices and annotations to R), and an object format converter (between the widely used scRNA-seq objects).

Introduction

In recent years, single-cell RNA sequencing (scRNA-seq) has emerged as a powerful tool to reveal cellular heterogeneity and fundamental characteristics of gene expression [1]. As a result, thousands of scRNA-seq datasets have been generated and uploaded to public repositories. Downloading and reanalyzing the existing scRNA-seq data provides an efficient choice to gain clues and new insights. For example, rare cell type identification [2] and pan-cancer scRNA-seq analysis [3] usually require a huge number of samples and cells, which is extremely expensive and time-consuming if not integrating the available scRNA-seq datasets. As well, exploring existing relevant scRNA-seq data can accelerate the progress of one's own research.

The existing scRNA-seq data are in three types: raw data (*sra*/*fastq*/*bam*), count matrix, and processed object (e.g. *rds* and *h5ad*), which are stored in different repositories. The raw data are mainly stored in Sequence Read Archive (SRA) [4] and European Nucleotide Archive (ENA) [5], the count matrices can be found in Gene Expression Omnibus (GEO) [6] and some scRNA-seq databases (e.g. UCSC Cell Browser [7] and PanglaoDB [8]), and a majority of processed objects are provided by GEO, Zenodo [9], Human Cell Atlas (HCA) [10], and CELLxGENE [11]. The diversity of data types and their distribution in specialized repositories make scRNA-seq data download difficult. Secondly, the wide array of available scRNA-seq protocols and preprocessing tools has led to differences in the data they produce and store. Representatively, there are differences between 10x Genomics (10x) [12] and Smart-seq2 [13] in terms of sequencing reads and output count matrix forms. How to process and load these

different types of downloaded files into a unified object that can be analyzed matter. Thirdly, the formats and programming languages of downloaded processed objects are varied due to the prosperity in scRNA-seq analysis tools, such as *SeuratObject* (*Seurat*, R) [14], *AnnData* (*Scanpy*, Python) [15], *CellDataSet*/*cell_data_set* (*Monocle2*/*3*, R) [16, 17], and *SingleCellExperiment* (*scater*, R) [18]. Diverse object formats and programming languages hinder the integration of scRNA-seq data and the interoperability between different analysis tools, thus the object format conversion is vital.

Current available tools mainly focus on downloading raw data (SRA and ENA) and count matrices (GEO) (Table 1). *ffq* [19] is designed to fetch sample metadata, including the download links, and therefore requires third-party tools to perform downloading. *GEOfastq* [20], *SRA-Toolkit* [21], *enaBrowserTools* [22], *fastq-dl* [23], *pysradb* [24], and *GEOfetch* [25] support downloading raw data from SRA and/or ENA. Among them, *GEOfastq*, *fastq-dl*, and *GEOfetch* can only download *fastq*, *sra*/*fastq*, and *sra* files respectively, and *GEOfetch* supports splitting *sra* into *fastq*/*bam* files. *GEOfastq*, *enaBrowserTools*, *fastq-dl* (version <1.2.0), and *pysradb* support downloading data via Aspera, while *fastq-dl* and *pysradb* parallelize the download process. *pysradb*, *GEOfetch*, and *GEOquery* [26] are able to download the supplementary files from GEO, and *GEOquery* can extract the count matrix from *ExpressionSet*. *rPanglaoDB* [27] can be used to download scRNA-seq count matrices and annotations from PanglaoDB, which only contains datasets originating from human and mouse, has relatively small sample numbers, and is no longer maintained. The above generalized tools, except

rPanglaoDB, support downloading raw data and/or count matrices from limited databases. And this download is regardless of whether the data are from scRNA-seq or bulk RNA-seq, thus lacking support for unique characteristics of scRNA-seq data (such as 10x-style *fastq* files and *bam* files with custom tags). *rPanglaoDB* specializes in downloading scRNA-seq count matrices and annotations, but the support is quite insufficient. As for format conversion, many tools have been developed for the same conversion stream. From *SeuratObject* to *AnnData* (Seu2AD), available tools are *SeuratDisk*, *sceasy*, and *scDIOR*, while the above tools and *schard* are suitable for the reverse stream (AD2Seu). From *SingleCellExperiment* to *AnnData* (SCE2AD), available tools are *sceasy*, *scDIOR*, and *zellkonverter*, while *scDIOR*, *zellkonverter*, and *schard* are suitable for the reverse stream (AD2SCE). Multiple available tools for the same stream confused the format conversion process, and currently lacking a benchmark of format conversion tools to guide the tool selection. Besides, no single tool can cover all the conversion streams between the widely used scRNA-seq objects.

To address the above issues, we present *GEfetch2R*, an R package to (i) fetch scRNA-seq/bulk RNA-seq raw data, count matrices, and processed objects from diverse public repositories; (ii) process the downloaded data, load the count matrices, annotations, and *rds* files to R (*SeuratObject*/*DESeqDataSet*), filter the *SeuratObject* based on cell metadata and genes, and dissect and extract the *RData* files; (iii) convert formats between the widely used scRNA-seq objects, and benchmark format conversion tools to guide the tool selection.

Materials and Methods

Download, process, and load raw data

With a given GEO accession as input, *GEfetch2R* can download raw data (*sra*/*fastq*/*bam*) from SRA and ENA (Supplementary Fig. S1). Firstly, *GEfetch2R* extracts all sample metadata under the given GEO accession, users can skip this step and provide a data frame containing the interested samples. The output sample metadata is used as input for subsequent raw data download. For *sra* files, *GEfetch2R* uses *prefetch* command to download them from SRA, and uses *ascp*, *download.file*, and *wget* commands in parallel to download them from ENA.

For *fastq* files in SRA, *GEfetch2R* uses *parallel-fastq-dump* (parallel), *fasterq-dump* (parallel), and *fastq-dump* commands to split the downloaded *sra* files into *fastq* files. If the data are from 10x, the *--split-files* parameter is added and at least two *fastq* files are generated. *GEfetch2R* automatically distinguishes read1 and read2 based on read length, and renames them according to the *CellRanger* required format. As for data from other scRNA-seq protocols or bulk RNA-seq, the *--split-3* parameter is used. For *fastq* files in ENA, *GEfetch2R* supports splitting the downloaded *sra* files as above, alternatively, if there are *fastq* files in ENA, *GEfetch2R* uses *ascp*, *download.file*, and *wget* commands in parallel to download them directly.

For *bam* files in SRA, if they are from 10x, *GEfetch2R* uses *prefetch* command with *--type TenX* parameter to download the original uploaded *bam* files directly to keep the custom tags required to reconstruct the original *fastq* files. As for *bam* files from other

scRNA-seq protocols or bulk RNA-seq, *GEfetch2R* uses *sam-dump* commands to split the downloaded *sra* files into *bam* files. For *bam* files in ENA, *GEfetch2R* uses *ascp*, *download.file*, and *wget* commands in parallel to download them directly. If *fastq* files are needed, *GEfetch2R* uses *bamtofastq* command developed by 10x to convert the downloaded 10x-generated *bam* files to *fastq* files, and uses *samtools* [28] command to convert the downloaded *bam* files generated by other scRNA-seq protocols or bulk RNA-seq to *fastq* files.

With prepared *fastq* files, if they are from 10x, *GEfetch2R* uses *CellRanger* to perform read alignment and feature counting (merge multiple runs of a sample). The output count matrix is then loaded to R using *Seurat*. If multiple count matrices are available, *GEfetch2R* merges multiple *SeuratObjects* if applicable. As for *fastq* files from Smart-seq2 or bulk RNA-seq, *GEfetch2R* uses *STAR* to perform read alignment and feature counting with *--quantMode GeneCounts* parameter (merge multiple runs of a sample). The output count matrix is then loaded to R using *DESeq2*.

To simplify the procedures from downloading raw data to loading to R, *GEfetch2R* provides a wrapper function *DownloadFastq2R*, which can extract all runs under a given GEO accession, automatically identify the RNA-seq type (10x Genomics scRNA-seq, Smart-seq2 scRNA-seq, and bulk RNA-seq) of each run, download *fastq* files directly from ENA, perform read alignment and feature counting using *CellRanger*/*STAR* (merge multiple runs of a sample), and load the results to R (*SeuratObject*/*DESeqDataSet*).

Download, process, and load count matrices and annotations

As in Supplementary Fig. S1, *GEfetch2R* provides two ways to access count matrices: with given accessions or links as input (GEO, UCSC Cell Browser, and PanglaoDB) and with filtered metadata as input (UCSC Cell Browser and PanglaoDB). The first way can be used to download specific datasets. For the latter way, *GEfetch2R* first extracts detailed metadata of all datasets, such as description, source/tissue, organism, protocol, and related publication. Users can filter datasets based on these attributes, and the filtered metadata is used as subsequent input. This way can be used to download datasets with similar characteristics in bulk.

When fetching count matrices from GEO, *GEfetch2R* tries to extract the count matrix from *ExpressionSet* first. If the extracted count matrix is empty or contains non-integer values, *GEfetch2R* generates the count matrix from supplementary files. For supplementary files in *CellRanger* output format, *GEfetch2R* automatically categorizes the downloaded files based on sample names. For supplementary files composed of the count matrix of every single-cell/bulk sample, *GEfetch2R* creates a count matrix containing all cells/samples. Users can also add *down.supp = TRUE* parameter in *ParseGEO* function to generate the count matrix directly from supplementary files. Besides, *GEfetch2R* also provides function *ExtractGEOMeta* to retrieve detailed sample metadata from the supplementary file or the GEO platform (user-provided when uploading). For UCSC Cell Browser, *GEfetch2R* accesses count matrices online instead of downloading them to save time and disk usage. In addition to count matrices, UCSC Cell Browser contains diverse annotations, such as cell type annotation, cell type

composition, and dimensionality reduction coordinates, *GEfetch2R* supports extracting all these annotations. For PanglaoDB, similar to UCSC Cell Browser, it also contains various annotations (e.g. cell type annotation and cell type composition), *GEfetch2R* uses *rPanglaoDB* to extract count matrices and corresponding annotations.

With available scRNA-seq count matrices and various annotations, *GEfetch2R* loads the count matrices to R using *Seurat*, adds available annotations to the *SeuratObjects* (UCSC Cell Browser and PanglaoDB), filters the *SeuratObjects* based on cell metadata and genes (UCSC Cell Browser and PanglaoDB), and merges *SeuratObjects* if applicable. For the bulk RNA-seq count matrix containing all samples, *GEfetch2R* loads it to R using *DESeq2* (GEO).

Download and load processed objects

Similar to downloading count matrices, *GEfetch2R* also provides two ways to access processed objects: with given accessions or links as input (GEO, Zenodo, CELLxGENE, and HCA) and with filtered metadata as input (CELLxGENE and HCA) (Supplementary Fig. S1).

For processed objects in GEO/Zenodo, with provided accession/DOIs, the index of the supplementary file (GEO), and the file extensions (e.g. *rds* and *h5ad*), *GEfetch2R* downloads the corresponding processed objects. For processed objects in CELLxGENE, *GEfetch2R* provides two methods to download them. *GEfetch2R* uses *cellxgene-census* [29] developed by the CELLxGENE team to efficiently access the cloud-hosted Census single-cell data and filter the data based on cell metadata and

genes. However, some newly uploaded data that can be explored on the web page may be missing due to the delay of cloud hosting (e.g. the stable Census release is 2025-01-30 when the current date is 2025-02-26). To overcome this, *GEfetch2R* takes advantage of CZ CELLxGENE Discover API to get the download links of processed objects (*rds* and *h5ad* files) directly, the same as clicking the download button on the web page. For processed objects in HCA, with links/filtered metadata and provided file extensions (e.g. *rds* and *h5ad*), *GEfetch2R* downloads the corresponding processed objects.

With processed objects in *rds* format, *GEfetch2R* loads them (*SeuratObjects*) to R, filters the *SeuratObjects* based on cell metadata and genes (CELLxGENE), and merges *SeuratObjects* if applicable. With processed objects in *RData* format, *GEfetch2R* dissects the elements within, extracts count matrices and metadata from standard (*SeuratObject*, *SingleCellExperiment*, *cell_data_set*, *CellDataSet*, *DESeqDataSet*, *DGEList*) or non-standard objects, and loads them to R if applicable.

Benchmark format conversion tools

To inspect the information kept after format conversion, we used the pbmc3k dataset analyzed by the standard *Seurat* and *Scanpy* workflow. The *SingleCellExperiment* used to inspect the information kept from *SingleCellExperiment* to *AnnData* was generated by *zellkonverter* (convert *AnnData* to *SingleCellExperiment*). The detailed code and output used to evaluate the retained information are provided in Supplementary Note.

To examine the running time and scalability of format conversion tools, we used *GEfetch2R* to select and download five processed objects (both *rds* and *h5ad* files) with

more than one million cells from CELLxGENE (Supplementary Table S1), then subsampled them to varied cell numbers: 1.2 million (1.2M, two objects), 1 million (1M), 800,000 (800K), 600,000 (600K), 500,000 (500K), 300,000 (300K), 200,000 (200K), 100,000 (100K), 80,000 (80K), 60,000 (60K), 50,000 (50K), 30,000 (30K), 20,000 (20K), 10,000 (10K). Each format conversion tool was executed on all the five object sets (64 objects in total). For the same object set, we ensured consistency in the type of count matrices converted between different tools to avoid potential bias. All format conversion tools were executed on a server with one Intel(R) Xeon(R) Gold 5220R CPU, 512GB RAM, and CentOS 7.5.1804 operating system. The running time (elapsed time) was recorded using *system.time* command in R. To ensure the independence of each run, all commands are run in linear order on the Linux shell as R scripts.

Format conversion between scRNA-seq objects

GEfetch2R uses *Seurat* to convert formats between *SeuratObject* and *SingleCellExperiment*, uses *Seurat* and *SeuratDisk* to convert formats between *SeuratObject* and *loom*, uses *Seurat* and *SeuratWrappers* to convert formats between *SeuratObject* and *CellDataSet*/*cell_data_set*, and uses *LoomExperiment* to convert formats between *SingleCellExperiment* and *loom*. For format conversion between *AnnData* and *SeuratObject*/*SingleCellExperiment*, all the tools used for benchmarking are available in *GEfetch2R*.

Results

Overview of *GEfetch2R*

GEfetch2R supports downloading all the three types of scRNA-seq data: raw data, count matrix, and processed object (Fig. 1). The databases corresponding to each data type, number of datasets/species/cells available, support formats, returned values, and other key features are summarized in Table 2. For fetching raw data, with a given GEO accession as input, *GEfetch2R* downloads *sra*, *fastq*, and *bam* files from SRA and ENA. In particular, the parameters for downloading 10x-related *fastq* and *bam* files have been optimized, such as downloading and formatting the *fastq* files to the *CellRanger* required format and downloading *bam* files with original tags. With downloaded files, *GEfetch2R* processes them and loads the output to R (*SeuratObject* for 10x-generated data, *DESeqDataSet* for Smart-seq2 or bulk RNA-seq data). For fetching count matrices, with given accessions/links (GEO, UCSC Cell Browser, and PanglaoDB) or filtered metadata (UCSC Cell Browser and PanglaoDB) as input, *GEfetch2R* accesses count matrices and various annotations, loads them to R, and extracts subset based on cell metadata and genes (UCSC Cell Browser and PanglaoDB). For fetching processed objects, with given accessions/links (GEO, Zenodo, CELLxGENE, and HCA) or filtered metadata (CELLxGENE and HCA) as input, *GEfetch2R* downloads processed objects in given file extensions, loads *rds* files (*SeuratObjects*) to R, extracts subset based on cell metadata and genes (CELLxGENE), and dissects and extracts the *RData* files. The processed objects in other formats can be loaded to R/Python through format conversion.

To enable the interoperability between scRNA-seq analysis tools and the integration of processed objects in diverse formats, *GEfetch2R* supports converting formats (i) between *SeuratObject* and *AnnData*, *SingleCellExperiment*, *CellDataSet*/*cell_data_set*, *loom*; (ii) between *SingleCellExperiment* and *loom*; (iii) between *SingleCellExperiment* and *AnnData*. Moreover, as several tools have been developed for the same conversion stream between *AnnData* and *SeuratObject*/*SingleCellExperiment*, we benchmarked their performance in terms of information kept, usability, running time, and scalability.

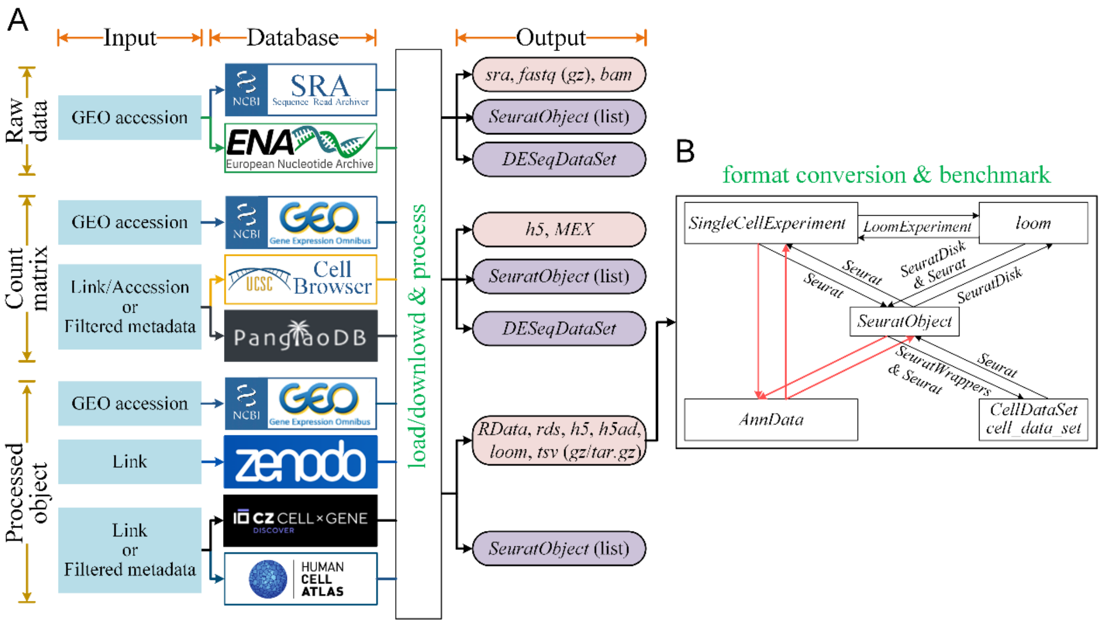

Figure 1: Overview of *GEfetch2R*. (A) Fetching and loading various data from public repositories to R. The download of raw data, count matrix, and processed object is indicated by ochre borders (horizontal). The input, supported databases, and final output are indicated by orange borders (vertical). The boxes in purple indicate R objects, in light pink indicate downloaded file formats. *MEX*: Market Exchange Format. (B) Format conversions supported by *GEfetch2R*. The boxes indicate scRNA-seq objects, the directional arrows between boxes indicate conversion streams between objects, the

texts above arrows represent the tool used for the conversion, the red directional arrows without texts indicate conversion streams that are benchmarked.

Application of *GEfetch2R* in COVID-19 scRNA-seq atlas exploration

Coronavirus disease 2019 (COVID-19) is a highly contagious disease caused by severe acute respiratory syndrome coronavirus 2, with symptoms ranging from mild to severe [30]. Depicting the dynamic immune responses across symptom severities at the single-cell level is an effective method to understand COVID-19 progression. To demonstrate the utility of *GEfetch2R*, we applied it to download and explore all T cells of a COVID-19 scRNA-seq atlas [30] from UCSC Cell Browser. As shown in Fig. 2A, there are twelve T cell subtypes, including six subtypes of $CD4^+$ T cells, three subtypes of $CD8^+$ T cells, and three subtypes of natural killer T (NKT) cells. The cell type composition analysis across four conditions (healthy donor (HD), moderate, severe, and convalescent (conv)) revealed that compared with HDs, the proportions of four $CD4^+$ T subtypes ($CD4^+$ naive, $CD4^+$ memory, $CD4^+$ effector memory, and regulatory T (Treg)) and $CD8^+$ naive subtype were significantly decreased in COVID-19 patients, and the proportion of $CD4^+$ naive subtype was still significantly reduced in COVID-19 conv samples (Fig. 2B-C). Meanwhile, the proportions of $CD4^+$ effector-GNLY, $CD8^+$ effector-GNLY, NKT CD56, and NKT CD160 subtypes were significantly increased in COVID-19 patients, and the $CD4^+$ effector-GNLY subtype was nearly absent in HDs but highly enriched in moderate, severe, and conv samples (Fig. 2B-C). The apoptosis scoring results showed that T cells in COVID-19 patients generally had significantly increased apoptosis scores (Fig. 2D).

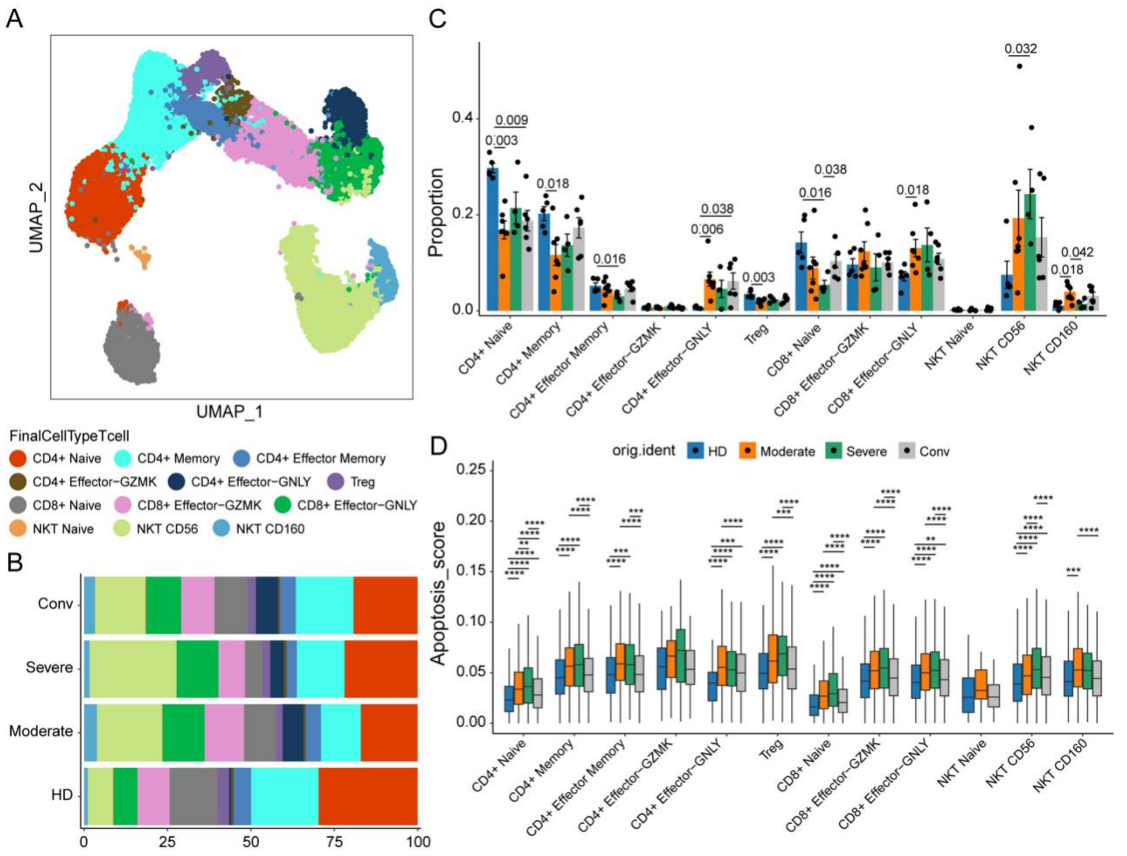

Figure 2: Application of *GEfetch2R* in COVID-19 scRNA-seq atlas exploration. (A) UMAP embedding of all T cells. Clusters in different colors represent T cell subtypes. (B) The proportion of each cell type across HD, moderate, severe, and conv samples. (C) Differential cell type composition analysis. All differences with $P < 0.05$ are labeled; two-sided unpaired Mann–Whitney U-test was used for analysis. (D) The apoptosis scoring results. All differences with Bonferroni adjusted $P < 0.01$ are indicated. **$P < 0.01$; ***$P < 0.001$; ****$P < 0.0001$; using two-sided unpaired Dunn's test. NKT: natural killer T; Treg: regulatory T.

Benchmark of format conversion tools

Information kept

We evaluated and ranked the information kept of format conversion tools in terms of count matrix and annotation. Fig. 3A and Supplementary Note show our rank reasoning and the overall information kept rank of each tool. In Seu2AD, *scDIOR* can preserve all the three count matrices and more annotations, thus ranking first. *SeuratDisk* can keep two of the three count matrices and more annotations, thus ranking second. While *sceasy* keeps only one count matrix and the least annotations, thus ranking last. In SCE2AD, *zellkonverter* and *scDIOR* both keep all the three count matrices, while *zellkonverter* preserves the most comprehensive annotations, thus *zellkonverter* ranks first. *sceasy* keeps only one count matrix and less comprehensive annotations, resulting in the lowest information kept rank. In AD2Seu, *scDIOR*, *sceasy*, and *SeuratDisk* can keep two of the three count matrices, which is more than *schard*. And, *scDIOR* preserves the most comprehensive annotations, thus ranking first. Both *schard* and *sceasy* retain the least annotations, thus *schard* ranks last. In AD2SCE, *zellkonverter* can preserve two of the three count matrices and the most comprehensive annotations, thus ranking first. While both *schard* and *scDIOR* keep only one count matrix, and *schard* retains the least annotations, thus *schard* ranks last.

A

Conversion	Tool	Count matrix	Annotation	Notes	Information rank
Seu2AD	SeuratDisk	data, counts/scale.data(conditional)	obs (meta.data), var (meta.features), uns, obsm (reductions), obsp (graphs), layers (assays)	the obsp only contains distances, not connectivities	2
	sceasy	counts/data/scale.data	obs (meta.data), var (meta.features), obsm (reductions)		3
	scDIOR	counts, data, scale.data(conditional)	obs (meta.data), var (meta.features), obsm (reductions), layers (assays), obsp (graphs)		1
SCE2AD	sceasy	counts/data/scale.data	obs (colData), var and varm (rowData), obsm (reducedDim)		3
	scDIOR	counts, data, scale.data	obs (colData), var (rowData), obsm (reducedDim), layers (assays)		2
	zellkonverter	counts, data, scale.data	obs (colData), var and varm(rowData), obsm (reducedDim), layers (assays), uns (metadata), obsp (colPairs)		1
AD2Seu	SeuratDisk	scale.data, counts/data(conditional)	meta.data (obs), meta.features (var), reductions (obsm, varm), misc (uns)	the misc is incomplete, only includes pca and umap	2
	sceasy	scale.data, counts/data(conditional)	meta.data (obs), meta.features (var), reductions (obsm)	the meta.features is incomplete	3
	scDIOR	scale.data, counts/data(conditional)	meta.data (obs), meta.features (var), reductions (obsm), assays (layers), graphs (obsp)	the meta.features is incomplete	1
	schard	counts/data/scale.data	meta.data (obs), meta.features (var), reductions (obsm)		4
AD2SCE	scDIOR	counts/data/scale.data	colData (obs), rowData (var), reducedDim (obsm), assays (layers)		2
	zellkonverter	scale.data, counts/data(conditional)	colData (obs), rowData (var, varm), reducedDim (obsm), assays (layers), metadata (uns), colPairs (obsp)		1
	schard	counts/data/scale.data	colData (obs), rowData (var), reducedDim (obsm)		3

B

Conversion	Tool	Programming language	Dependency	Availability	Documanetation	Support	Compatibility	Others	Usability rank
Seu2AD	SeuratDisk	R		GitHub, Conda	README, webpage	no Q&A	excellent	two steps, generate intermediate file	2
	sceasy	R	python env	GitHub, Conda	README	no Q&A	excellent		3
	scDIOR	R, Python		GitHub	README	no Q&A	excellent	generate h5 instead of h5ad file	1
SCE2AD	sceasy	R	python env	GitHub, Conda	README	no Q&A	excellent		3
	scDIOR	R, Python		GitHub	README	no Q&A	excellent	generate h5 instead of h5ad file	1
	zellkonverter	R	python env	GitHub, Conda, Bioconductor	webpage	active Q&A	excellent		2
AD2Seu	SeuratDisk	R		GitHub, Conda	README, webpage	no Q&A	fail (failure on test and converted datasets)	two steps, generate intermediate file	3
	sceasy	R	python env	GitHub, Conda	README	no Q&A	good (failure on test datasets, but success on zellkonverter converted datasets)		2
	scDIOR	R, Python	python env	GitHub	README	no Q&A	fail (failure on test and converted datasets)		4
	schard	R		GitHub	README	active Q&A	excellent		1
AD2SCE	scDIOR	R, Python	python env	GitHub	README	no Q&A	excellent		3
	zellkonverter	R	python env	GitHub, Conda, Bioconductor	webpage	active Q&A	excellent		2
	schard	R		GitHub	README	active Q&A	excellent		1

Figure 3: The information kept and usability of format conversion tools. (A) The information kept of format conversion tools in terms of count matrix and annotation. In 'Count matrix' column, 'counts' represents raw count matrix, 'data' represents normalized data matrix, 'scale.data' represents scaled data matrix. In 'Annotation' column, the text outside/inside parentheses represents the data slot of the source/target object. (B) The usability of format conversion tools in terms of programming language, dependency, availability, documentation, support, and compatibility. Q&A: question and answer. The tool with the highest rank is marked in green.

Usability

We evaluated and ranked the usability of format conversion tools in terms of programming language, dependency, availability, documentation, support, and compatibility. Fig. 3B shows our rank reasoning and the overall usability rank of each tool. In Seu2AD, *sceasy* requires an additional Python environment, resulting in the lowest usability rank. *SeuratDisk* and *scDIOR* have comparable usability, but *scDIOR* can be used in both R and Python platforms, thus having a higher usability rank. In SCE2AD, *scDIOR* is the only tool that doesn't require an additional Python environment and can be used in both R and Python platforms, resulting in the highest usability rank. *zellkonverter* is hosted on Bioconductor and has active questions and answers (Q&As), thus ranking second. In AD2Seu, *schard* doesn't require an additional Python environment, has active Q&As, and is the only tool that runs successfully on all test data. Among the remaining three tools, *sceasy* runs successfully on *zellkonverter* converted *AnnData*, while the other tools still fail. Based on the above results, *schard* ranks first and *sceasy* ranks second in usability, and the subsequent running time is recorded on *zellkonverter* converted *AnnData*. In AD2SCE, *schard* has active Q&As and is the only tool that doesn't require an additional Python environment, resulting in the highest usability rank. *zellkonverter* and *scDIOR* have comparable usability, but the former is slightly better. In detail, *zellkonverter* is hosted on Bioconductor and has active Q&As, while *scDIOR* can be used in both R and Python platforms.

Running time and scalability

In Supplementary Table S2, we summarized the running time of the format conversion tools for converting between *AnnData* and *SeuratObject*/*SingleCellExperiment* on five object sets (64 objects in total). Fig. 4 shows that in AD2SCE, *schard* is always the fastest tool across all the five object sets, while *zellkonverter* is the slowest tool in two of the five object sets. In AD2Seu, when the cell number is smaller than 200K, *schard* is faster than *sceasy* or has comparable speed, but *sceasy* is significantly faster than *schard* when the cell number exceeds 200K. This alternation means that *sceasy* has better scalability than *schard*. In SCE2AD, *zellkonverter* is always the fastest tool across all the five object sets, while *sceasy* and *scDIOR* are the slowest tools in two of the five object sets. In Seu2AD, across all the five object sets, *sceasy* is always the fastest tool, while *SeuratDisk* is always the slowest tool.

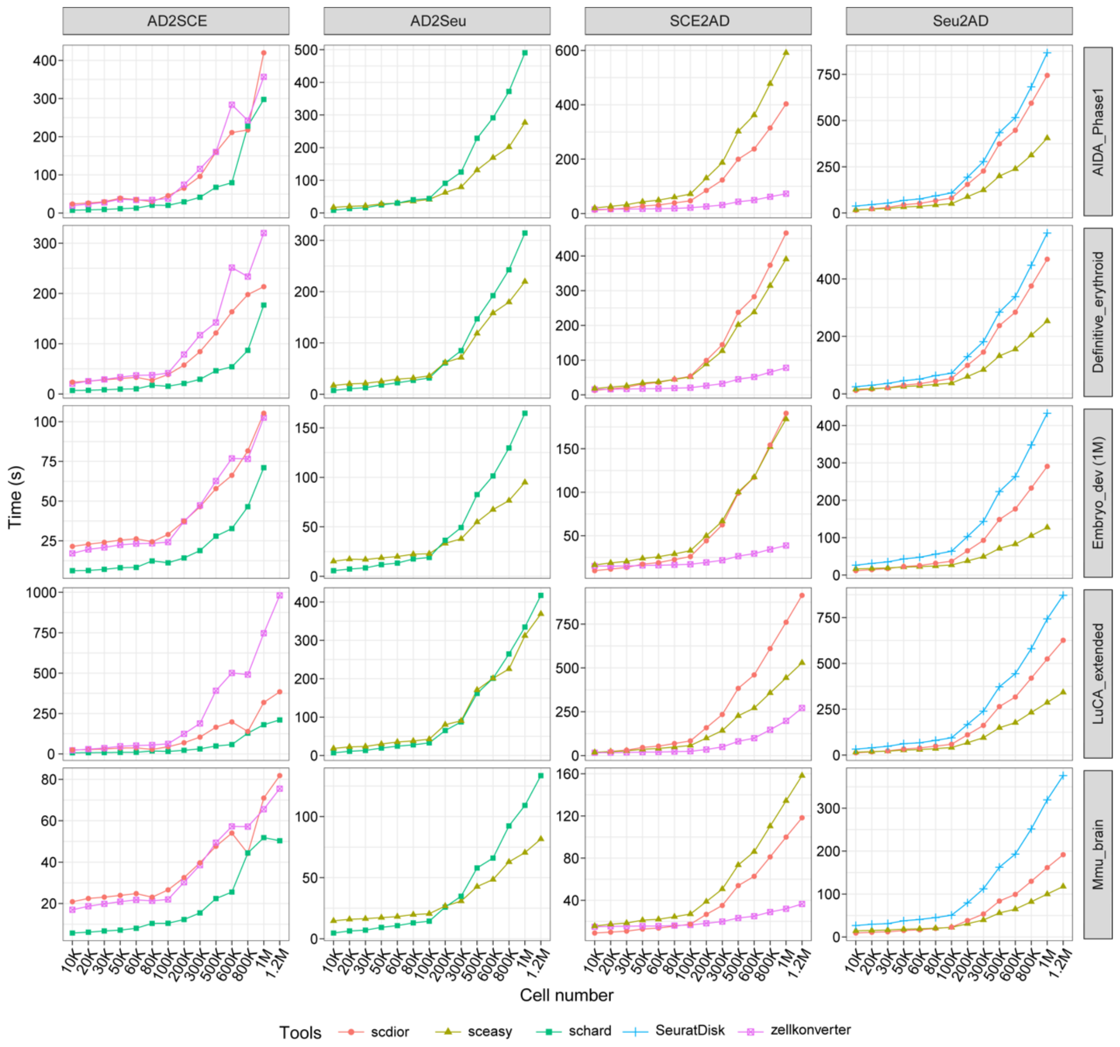

Figure 4: The running time of format conversion tools. There are twenty subplots, distributed in four rows and five columns. Each row represents an object set. Each column represents the same conversion stream. *x*-axis means the cell number (K: thousand, M: million), *y*-axis means the running time.

Comparison of *GEfetch2R* with other similar tools

Table 1 shows a feature comparison of *GEfetch2R* with other tools that are capable of downloading data from public repositories. *GEfetch2R* distinguishes itself in the following aspects. Firstly, *GEfetch2R* supports downloading the most diverse data types

(raw data, count matrix, and processed object), and each data type can be downloaded from multiple public repositories. This benefits users in collecting data more comprehensively and choosing appropriate download data types according to different needs. For quick data exploration, downloading processed objects is a good choice, whereas for fine data integration to draw conclusions, downloading raw data or count matrices can help reduce the bias. Secondly, *GEfetch2R* adopts multiple methods to speed up downloading, including Aspera support, parallel downloading *fastq* files, and parallel splitting *sra* files into *fastq* files. Thirdly, *GEfetch2R* supports downloading data of both scRNA-seq and bulk RNA-seq, and considers the characteristics of data generated by different protocols. For 10x-generated data, *GEfetch2R* downloads and formats the *fastq* files to the *CellRanger* required format, downloads *bam* files with original tags, converts *bam* files to *fastq* files using *bamtofastq*, and aligns *fastq* files to the reference genome using *CellRanger*. For Smart-seq2 and bulk RNA-seq data, *GEfetch2R* converts *bam* files to *fastq* files using *samtools* and aligns *fastq* files to the reference genome using *STAR*. This expands the scope of use of *GEfetch2R* and makes the data it downloads easier to use. Fourthly, besides data download, *GEfetch2R* can process the downloaded data, load the count matrices, annotations, and *rds* files to R (*SeuratObject*/*DESeqDataSet*), extract a subset of the *SeuratObject* based on cell metadata and genes, and dissect and extract the *RData* files. This greatly distinguishes *GEfetch2R* from other tools, which are pure data downloaders. Lastly, *GEfetch2R* provides the most comprehensive object format conversions, and benchmarks the format conversion tools for converting between *AnnData* and

SeuratObject/*SingleCellExperiment*. This enables users to integrate multiple objects in diverse formats, bridges the widely used scRNA-seq analysis tools, and guides the selection of format conversion tools.

Conclusions and Discussions

GEfetch2R is an R package dedicated to facilitating researchers to access and explore the existing gene expression data from various public repositories. As a data downloader, *GEfetch2R* supports downloading the most diverse scRNA-seq data types, including raw data (SRA and ENA), count matrix (GEO, UCSC Cell Browser, and PanglaoDB), and processed object (GEO, Zenodo, CELLxGENE, and HCA). Besides the data download ability, *GEfetch2R* can process the downloaded data, load the count matrices, annotations, and *rds* files to R (*SeuratObject*/*DESeqDataSet*), extract a subset of the *SeuratObject* based on cell metadata and genes, and dissect and extract the *RData* files. Furthermore, to enable the integration of scRNA-seq data and the interoperability between different analysis tools, *GEfetch2R* provides the most comprehensive format conversions between different scRNA-seq objects, including *SeuratObject*, *AnnData*, *SingleCellExperiment*, *CellDataSet*/*cell_data_set*, and *loom*. In particular, *GEfetch2R* benchmarks the format conversion tools for converting between *SeuratObject*/*SingleCellExperiment* and *AnnData*. In Seu2AD, *scDIOR* has the best performance in terms of information kept and usability, and *sceasy* is always the fastest tool. In SCE2AD, *zellkonverter* has the highest information kept rank, *scDIOR* is the best in usability, and *zellkonverter* is consistently the fastest tool. In AD2Seu, *scDIOR* has the best performance in terms of information kept, *schard* is the best in usability,

and *sceasy* has better scalability than *schard*. In AD2SCE, *zellkonverter* has the highest information kept rank, *schard* is the best in terms of usability and speed.

In addition to fetching scRNA-seq data, *GEfetch2R* can also be used to download raw data (SRA and ENA) and count matrices (GEO) of bulk RNA-seq, process the downloaded data, and load output/downloaded count matrices to R (*DESeqDataSet*).

Currently, there are still several aspects of *GEfetch2R* that can be improved. Firstly, *GEfetch2R* only supports processing the Smart-seq2 and 10x-generated raw data, users can download the raw data generated by other scRNA-seq protocols using *GEfetch2R*, but the subsequent process is not available. Secondly, many widely used scRNA-seq databases are not supported by *GEfetch2R*, such as Single Cell Portal [31] and gEAR [32]. We will actively develop to support more scRNA-seq protocols and databases. Besides, since the APIs of supported databases may change, we will update *GEfetch2R* on time to ensure usage.

Availability and Requirements

- Project name: *GEfetch2R*
- Project homepage: https://github.com/showteeth/GEfetch2R
- Software documentation: https://showteeth.github.io/GEfetch2R
- Docker image: https://hub.docker.com/r/soyabean/gefetch2r
- Operating system(s): Platform independent
- Programming language: R

- Other requirements: R 2.10 or higher, Python 3.7.3 or higher (format conversion)
- License: GPL-3.0
- RRID: SCR_026714
- biotoolsID: gefetch2r

Additional Files

Supplementary Note. Step-by-step code and output showing information kept of format conversion tools.

Supplementary Fig. S1. The detailed workflow of *GEfetch2R*.

Supplementary Table S1. The datasets used for benchmarking format conversion tools.

Supplementary Table S2. The running time of the format conversion tools on five object sets.

Abbreviations

scRNA-seq: single-cell RNA sequencing; SRA: Sequence Read Archive; ENA: European Nucleotide Archive; GEO: Gene Expression Omnibus; HCA: Human Cell Atlas; 10x: 10x Genomics; Seu2AD: from *SeuratObject* to *AnnData*; AD2Seu: from *AnnData* to *SeuratObject*; SCE2AD: from *SingleCellExperiment* to *AnnData*; AD2SCE: from *AnnData* to *SingleCellExperiment*; K: thousand; M: million; COVID-19: Coronavirus disease 2019; NKT: natural killer T; HD: healthy donor; conv: convalescent; Treg: regulatory T; Q&As: questions and answers.

Acknowledgements

We thank SRA, ENA, GEO, PanglanDB, UCSC Cell Browser, Zenodo, CELLxGENE, and HCA for generously hosting the single-cell/bulk RNA-seq data and providing websites/APIs for users to access these data, and gratefully acknowledge all the data contributors. These are the cornerstones for the development of *GEfetch2R*.

Funding

This work was supported by the National Natural Science Foundation of China (No. 22050004); the start-up funds from Institute of Microbiology of Chinese Academy of Sciences; and the Chinese Academy of Sciences Project for Young Scientists in Basic Research (No. YSBR-111).

Author Contributions

Y.S., J.W., and J.G. contributed to the conception and design of *GEfetch2R*. Y.S. implemented and benchmarked *GEfetch2R*. J.W. and J.G. provided supervision and secured funding. All authors prepared and approved the manuscript.

Data Availability

The datasets used in this study are freely available from UCSC Cell Browser ("COVID-19 Immunological Response"/"T-cells"), *SeuratData* (built-in dataset: pbmc3k.final) [33], 10x Genomics (3k PBMCs from a healthy donor), and CELLxGENE (Supplementary Table S1). All scripts used for the COVID-19 scRNA-seq atlas exploration and benchmark of format conversion tools, including downloading,

processing, subsampling, running, and visualizing, are available in the GitHub repository [34]. Snapshots of the code and data are available in Software Heritage [35]. All supporting data are available in the *GigaScience* repository, GigaDB [36].

Competing Interests

The authors declare that they have no competing interests.

References

1. Haque A, Engel J, Teichmann SA, et al. A practical guide to single-cell RNA-sequencing for biomedical research and clinical applications. Genome Med. 2017;9(1):1-12. 10.1186/s13073-017-0467-4.
2. Cheng Y, Fan X, Zhang J, et al. A scalable sparse neural network framework for rare cell type annotation of single-cell transcriptome data. Commun Biol. 2023;6(1):545. 10.1038/s42003-023-04928-6.
3. Zheng L, Qin S, Si W, et al. Pan-cancer single-cell landscape of tumor-infiltrating T cells. Science. 2021;374(6574):abe6474. 10.1126/science.abe6474.
4. Katz K, Shutov O, Lapoint R, et al. The sequence read archive: a decade more of explosive growth. Nucleic Acids Res. 2022;50(D1):D387-D90. 10.1093/nar/gkab1053.
5. Burgin J, Ahamed A, Cummins C, et al. The European Nucleotide Archive in 2022. Nucleic Acids Res. 2022;51(D1):D121-D5. 10.1093/nar/gkac1051.
6. Barrett T, Wilhite SE, Ledoux P, et al. NCBI GEO: archive for functional genomics data sets—update. Nucleic Acids Res. 2012;41(D1):D991-D5. 10.1093/nar/gks1193.
7. Speir ML, Bhaduri A, Markov NS, et al. UCSC cell browser: visualize your single-cell data. Bioinformatics. 2021;37(23):4578-80. 10.1093/bioinformatics/btab503.
8. Franzén O, Gan L-M , Björkegren JL. PanglaoDB: a web server for exploration of mouse and human single-cell RNA sequencing data. Database. 2019;2019:baz046. 10.1093/database/baz046.
9. Zenodo. CERN, Genève. 2013. https://www.zenodo.org/. Accessed 11 February 2026.
10. Regev A, Teichmann SA, Lander ES, et al. The human cell atlas. elife. 2017;6:e27041. 10.7554/eLife.27041.
11. Megill C, Martin B, Weaver C, et al. cellxgene: a performant, scalable exploration platform for high dimensional sparse matrices. bioRxiv. 2021:2021.04.05.438318. 10.1101/2021.04.05.438318.
12. Zheng GX, Terry JM, Belgrader P, et al. Massively parallel digital transcriptional profiling of single cells. Nat Commun. 2017;8(1):14049. 10.1038/ncomms14049.
13. Picelli S, Faridani OR, Björklund ÅK, et al. Full-length RNA-seq from single cells using Smart-seq2. Nat Protoc. 2014;9(1):171-81. 10.1038/nprot.2014.006.
14. Satija R, Farrell JA, Gennert D, et al. Spatial reconstruction of single-cell gene expression data. Nat Biotechnol. 2015;33(5):495-502. 10.1038/nbt.3192.

15. Wolf FA, Angerer P , Theis FJ. SCANPY: large-scale single-cell gene expression data analysis. Genome Biol. 2018;19:1-5. 10.1186/s13059-017-1382-0.
16. Qiu X, Mao Q, Tang Y, et al. Reversed graph embedding resolves complex single-cell trajectories. Nat Methods. 2017;14(10):979-82. 10.1038/nmeth.4402.
17. Cao J, Spielmann M, Qiu X, et al. The single-cell transcriptional landscape of mammalian organogenesis. Nature. 2019;566(7745):496-502. 10.1038/s41586-019-0969-x.
18. McCarthy DJ, Campbell KR, Lun ATL, et al. Scater: pre-processing, quality control, normalization and visualization of single-cell RNA-seq data in R. Bioinformatics. 2017;33(8):1179-86. 10.1093/bioinformatics/btw777.
19. Gálvez-Merchán Á, Min KH, Pachter L, et al. Metadata retrieval from sequence databases with ffq. Bioinformatics. 2023;39(1) 10.1093/bioinformatics/btac667.
20. Pickering A. GEOfastq. https://github.com/alexvpickering/GEOfastq. Accessed 11 February 2026.
21. NCBI. SRA-Toolkit. https://github.com/ncbi/sra-tools. Accessed 11 February 2026.
22. ENA. enaBrowserTools. https://github.com/enasequence/enaBrowserTools. Accessed 2 March 2025.
23. Petit R. fastq-dl. https://github.com/rpetit3/fastq-dl. Accessed 2 March 2025.
24. Choudhary S. pysradb: A Python package to query next-generation sequencing metadata and data from NCBI Sequence Read Archive. F1000Research. 2019;8:532. 10.12688/f1000research.18676.1.
25. Khoroshevskyi O, LeRoy N, Reuter VP, et al. GEOfetch: a command-line tool for downloading data and standardized metadata from GEO and SRA. Bioinformatics. 2023;39(3):btad069. 10.1093/bioinformatics/btad069.
26. Davis S , Meltzer PS. GEOquery: a bridge between the Gene Expression Omnibus (GEO) and BioConductor. Bioinformatics. 2007;23(14):1846-7. 10.1093/bioinformatics/btm254.
27. Osorio D, Kuijjer ML , Cai JJ. rPanglaoDB: an R package to download and merge labeled single-cell RNA-seq data from the PanglaoDB database. Bioinformatics. 2022;38(2):580-2. 10.1093/bioinformatics/btab549.
28. Li H, Handsaker B, Wysoker A, et al. The sequence alignment/map format and SAMtools. Bioinformatics. 2009;25(16):2078-9. 10.1093/bioinformatics/btp352.
29. Program CCS, Abdulla S, Aevermann B, et al. CZ CELLxGENE Discover: a single-cell data platform for scalable exploration, analysis and modeling of aggregated data. Nucleic Acids Res. 2024;53(D1):D886-D900. 10.1093/nar/gkae1142.
30. Zhang J-Y, Wang X-M, Xing X, et al. Single-cell landscape of immunological responses in patients with COVID-19. Nat Immunol. 2020;21(9):1107-18. 10.1038/s41590-020-0762-x.
31. Tarhan L, Bistline J, Chang J, et al. Single Cell Portal: an interactive home for single-cell genomics data. bioRxiv. 2023:2023.07.13.548886. 10.1101/2023.07.13.548886.
32. Orvis J, Gottfried B, Kancherla J, et al. gEAR: Gene Expression Analysis Resource portal for community-driven, multi-omic data exploration. Nat Methods. 2021;18(8):843-844. 10.1038/s41592-021-01200-9.
33. satijalab. SeuratData. https://github.com/satijalab/seurat-data. Accessed 11 February 2026.
34. Song Y. Code and data used for exploration and benchmark. https://github.com/showteeth/GEfetch2R/tree/main/man/benchmark. Accessed 23 March 2026.

35. Song Y. An archival copy of GEfetch2R. https://archive.softwareheritage.org/browse/origin/directory/?origin_url=https://github.com/showteeth/GEfetch2R. Accessed 23 March 2026.

36. Song Y, Wang J, Gao J. Supporting data for "GEfetch2R: fetching single-cell/bulk RNA-seq data from public repositories to R and benchmarking the subsequent format conversion tools" GigaScience Database. https://doi.org/10.5524/102815.

Table 1 Comparison of GEfetch2R with other similar tools

Click here to access/download;Table;Table 1.pdf

Table 1: Comparison of *GEfetch2R* with other similar tools

	GEfetch2R	*ffq*	*GEOfastq*	*SRA-Toolkit*	*enaBrowserTools*	*fastq-dl*	*pysradb*	*GEOfetch*	*GEOquery*	*rPanglaoDB*
**Programming language**	R	Python	R	C	Python	Python	Python	Python	R	R
**Accepted accession**	GEO	SRA, ENA, GEO	GEO	SRA, ENA	SRA, ENA	SRA, ENA	SRA, ENA, GEO	GEO	GEO	-
**Supported database**										
raw data (*sra* /*fastq* /*bam*)	SRA, ENA	-	ENA	SRA	ENA	SRA, ENA	SRA, ENA	SRA	-	-
count matrix	GEO, PanglaoDB, UCSC Cell Browser	-	-	-	-	-	GEO	GEO	GEO	PanglaoDB
processed object	GEO, Zenodo, CELLxGENE, Human Cell Atlas	-	-	-	-	-	-	-	-	-
**Downloaded data**										
metadata	+	+	+	-	+	+	+	+	+	+
raw data (*sra* /*fastq* /*bam*)	+++	-	*fastq*	+++	+++	*sra* , *fastq*	+++	*sra*	-	-
count matrix	+++	-	-	-	-	-	+	+	+	+
processed object	+++	-	-	-	-	-	-	-	-	-
**Aspera support**	Yes	-	Yes	-	Yes	-[a]	Yes	-	-	-
**Parallel download**	Yes	-	-	-	-	Yes	Yes	-	-	-
**Process downloaded data**										
sra to *fastq*	*fastq-dump* , *fasterq-dump* , *parallel-fastq-dump* [b]	-	-	*fastq-dump* , *fasterq-dump*	-	*fasterq-dump*	-	*fasterq-dump*	-	-
sra to *bam*	*sam-dump*	-	-	*sam-dump*	-	-	-	*sam-dump*	-	-
bam to *fastq*	*bamtofastq* (10x) *samtools* (others)	-	-	-	-	-	-	-	-	-
format *fastq* (10x)	Yes	-	-	-	-	-	-	-	-	-
mapping	*CellRanger* , *STAR*	-	-	-	-	-	-	-	-	-
**Load to R**										
DESeq2	Yes	-	-	-	-	-	-	-	*MAList, ExpressionSet*	-
Seurat	Yes	-	-	-	-	-	-	-	-	Yes
merge *SeuratObjects*	Yes	-	-	-	-	-	-	-	-	Yes

a: *fastq-dl* old version (<1.2.0) support Aspera
b: Parameters optimized for 10x Genomics data

Table 2 The databases supported corresponding to each data type Click here to access/download;Table;Table 2.pdf

Table 2: The databases supported corresponding to each data type

RNA-seq type	Data type	Download mode	Database	Dataset	Species	Cell	Support format	Returned value (non-NULL)	Annotation	Subset	Parallel	Others
scRNA-seq	raw data	given accession	SRA, ENA	unlimited	unlimited	unlimited	*fastq* (*gz*), *sra*, *bam*	*SeuratObject* (10x Genomics) / *DESeqDataSet* (Smart-seq2), failed sample metadata	sample metadata	/	Yes	multiple download and splitting methods, adapt to different scRNA-seq protocols, download status check, convert *bam* to *fastq*, merge multiple runs of a sample
	count matrix	given accession	GEO	unlimited	unlimited	unlimited	*csv*, *tsv*, *txt*, *tab*, *xlsx*, *xls*, *h5*, *MEX*[a] (*gz*/*tar.gz*)	*SeuratObject*	sample metadata	/	/	generate count matrix from supplementary files, adapt to multiple feature counting tools
		given accession, filtered metadata	PanglaoDB	1,368	Human/Mouse	5,586,348	/	*SeuratObject*	sample and cell metadata	cell type, gene	/	summarize metadata, extract cell type composition
		given link, filtered metadata	UCSC Cell Browser	1,427	34	141,087,666	/	*SeuratObject*	sample and cell metadata	cell metadata, gene	/	summarize metadata, extract cell type composition and dimensionality reduction coordinates
	processed object	given accession	GEO	unlimited	unlimited	unlimited	*RData*, *rds*, *h5ad*, *loom* (*gz*/*tar.gz*)	*SeuratObject*	sample and cell metadata	/	/	dissect and extract the *RData* files
		given link	Zenodo	unlimited	unlimited	unlimited	unlimited	*SeuratObject*, failed doi information	cell metadata	/	Yes	md5 check, dissect and extract the *RData* files
		given link, filtered metadata	CELLxGENE	2,043	8	248,553,530	*rds*[b], *h5ad*	*SeuratObject*, failed sample metadata	sample and cell metadata	cell metadata, gene	Yes	download status check, summarize metadata, extract dimensionality reduction coordinates
		given link, filtered metadata	Human Cell Atlas	546	4	~71,054,879	*RData*, *rds*, *h5*, *h5ad*, *loom*, *tsv* (*gz*/*tar.gz*)	*SeuratObject*, failed sample metadata	sample and cell metadata	/	Yes	download status check, summarize metadata, dissect and extract the *RData* files
bulk RNA-seq	raw data	given accession	SRA, ENA	unlimited	unlimited	unlimited	*fastq* (*gz*), *sra*, *bam*	*DESeqDataSet*, failed sample metadata	sample metadata	/	Yes	multiple download and splitting methods, download status check, convert *bam* to *fastq*, merge multiple runs of a sample
	count matrix	given accession	GEO	unlimited	unlimited	unlimited	*csv*, *tsv*, *txt*, *tab*, *xlsx*, *xls* (*gz*/*tar.gz*)	*DESeqDataSet*	sample metadata	/	/	generate count matrix from supplementary files, adapt to multiple feature counting tools

a: *MEX* stands for Market Exchange Format (barcodes.tsv.gz, features.tsv.gz/genes.tsv.gz, matrix.mtx.gz).
b: CELLxGENE has deprecated *Seurat* downloads since versions after 2025 (earlier *Seurat* downloads continue to be available). *GEfetch2R* has stored *Seurat* download links in May 2024.

Table 1 Click here to access/download;Table;Table 1.xlsx

Table 1: Comparison of *GEfetch2R* with other similar tools

	GEfetch2R	*ffq*	*GEOfastq*	*SRA-Toolkit*	*enaBrowserTools*	*fastq-dl*	*pysradb*	*GEOfetch*	*GEOquery*	*rPanglaoDB*
**Programming language**	R	Python	R	C	Python	Python	Python	Python	R	R
**Accepted accession**	GEO	SRA, ENA, GEO	GEO	SRA, ENA	SRA, ENA	SRA, ENA	SRA, ENA, GEO	GEO	GEO	-
**Supported database**										
raw data (*sra*/*fastq*/*bam*)	SRA, ENA	-	ENA	SRA	ENA	SRA, ENA	SRA, ENA	SRA	-	-
count matrix	GEO, PanglaoDB, UCSC Cell Browser	-	-	-	-	-	GEO	GEO	GEO	PanglaoDB
processed object	GEO, Zenodo, CELLxGENE, Human Cell Atlas	-	-	-	-	-	-	-	-	-
**Downloaded data**										
metadata	+	+	+	-	+	+	+	+	+	+
raw data (*sra*/*fastq*/*bam*)	+++	-	*fastq*	+++	+++	*sra*, *fastq*	+++	*sra*	-	-
count matrix	+++	-	-	-	-	-	+	+	+	+
processed object	+++	-	-	-	-	-	-	-	-	-
**Aspera support**	Yes	-	Yes	-	Yes	-[a]	Yes	-	-	-
**Parallel download**	Yes	-	-	-	-	Yes	Yes	-	-	-
**Process downloaded data**										
sra to *fastq*	*fastq-dump*, *fasterq-dump*, *parallel-fastq-dump*[b]	-	-	*fastq-dump*, *fasterq-dump*	-	*fasterq-dump*	-	*fasterq-dump*	-	-
sra to *bam*	*sam-dump*	-	-	*sam-dump*	-	-	-	*sam-dump*	-	-
bam to *fastq*	*bamtofastq* (10x) *samtools* (others)	-	-	-	-	-	-	-	-	-
format *fastq* (10x)	Yes	-	-	-	-	-	-	-	-	-
mapping	*CellRanger*, *STAR*	-	-	-	-	-	-	-	-	-
**Load to R**										
DESeq2	Yes	-	-	-	-	-	-	-	*MAList*, *ExpressionSet*	-
Seurat	Yes	-	-	-	-	-	-	-	-	Yes
merge *SeuratObjects*	Yes	-	-	-	-	-	-	-	-	Yes

a: *fastq-dl* old version (<1.2.0) support Aspera
b: Parameters optimized for 10x Genomics data

Click here to access/download;Table;Table 2.xlsx

Table 2: The databases supported corresponding to each data typ

RNA-seq type	Data type	Download mode	Database	Dataset	Species
scRNA-seq	raw data	given accession	SRA, ENA	unlimited	unlimited
	count matrix	given accession	GEO	unlimited	unlimited
		given accession, filtered metadata	PanglaoDB	1,368	Human/Mous
		given link, filtered metadata	UCSC Cell Browse	1,427	34
	processed object	given accession	GEO	unlimited	unlimited
		given link	Zenodo	unlimited	unlimited
		given link, filtered metadata	CELLxGENE	2,043	8
		given link, filtered metadata	Human Cell Atlas	546	4
bulk RNA-seq	raw data	given accession	SRA, ENA	unlimited	unlimited
	count matrix	given accession	GEO	unlimited	unlimited

a: *MEX* stands for Market Exchange Format (barcodes.tsv.gz, features.tsv.gz/genes.tsv.gz, m
b: CELLxGENE has deprecated *Seurat* downloads since versions after 2025 (earlier *Seurat*

›e

Cell	Support format	Returned value (non-NULL)	Annotation
unlimited	*fastq* (*gz*), *sra*, *bam*	*SeuratObject* (10x Genomics) */ DESeqDataSet* (Smart-seq2), failed sample metadata	sample metadata
unlimited	*csv*, *tsv*, *txt*, *tab*, *xlsx*, *xls*, *h5*, *MEX* [a] (*gz*/*tar.gz*)	*SeuratObject*	sample metadata
5,586,348	/	*SeuratObject*	sample and cell metadata
141,087,666	/	*SeuratObject*	sample and cell metadata
unlimited	*RData*, *rds*, *h5ad*, *loom* *(gz/tar.gz)*	*SeuratObject*	sample and cell metadata
unlimited	unlimited	*SeuratObject*, failed doi information	cell metadata
248,553,530	*rds* [b], *h5ad*	*SeuratObject*, failed sample metadata	sample and cell metadata
~71,054,879	*RData*, *rds*, *h5*, *h5ad*, *loom*, *tsv* (*gz*/*tar.gz)*	*SeuratObject*, failed sample metadata	sample and cell metadata
unlimited	*fastq (gz), sra, bam*	*DESeqDataSet*, failed sample metadata	sample metadata
unlimited	*csv*, *tsv*, *txt*, *tab*, *xlsx*, *xls* (*gz*/*tar.gz*)	*DESeqDataSet*	sample metadata

ıatrix.mtx.gz).
downloads continue to be available). *GEfetch2R* has stored *Seurat* download links in Ma

Subset	Paralle	Others
/	Yes	multiple download and splitting methods, adapt to different scRNA-seq protocols, download status check, convert *bam* to *fastq* , merge multiple runs of a sample
/	/	generate count matrix from supplementary files, adapt to multiple feature counting tools
cell type, gen	/	summarize metadata, extract cell type composition
cell metadata, gene	/	summarize metadata, extract cell type composition and dimensionality reduction coordinates
/	/	dissect and extract the *RData* files
/	Yes	md5 check, dissect and extract the *RData* files
cell metadata, gene	Yes	download status check, summarize metadata, extract dimensionality reduction coordinates
/	Yes	download status check, summarize metadata, dissect and extract the *RData* files
/	Yes	multiple download and splitting methods, download status check, convert *bam* to *fastq* , merge multiple runs of a sample
/	/	generate count matrix from supplementary files, adapt to multiple feature counting tools

ay 2024.

Figure 1 Overview of GEfetch2R

Click here to access/download;Figure;Figure 1.pdf

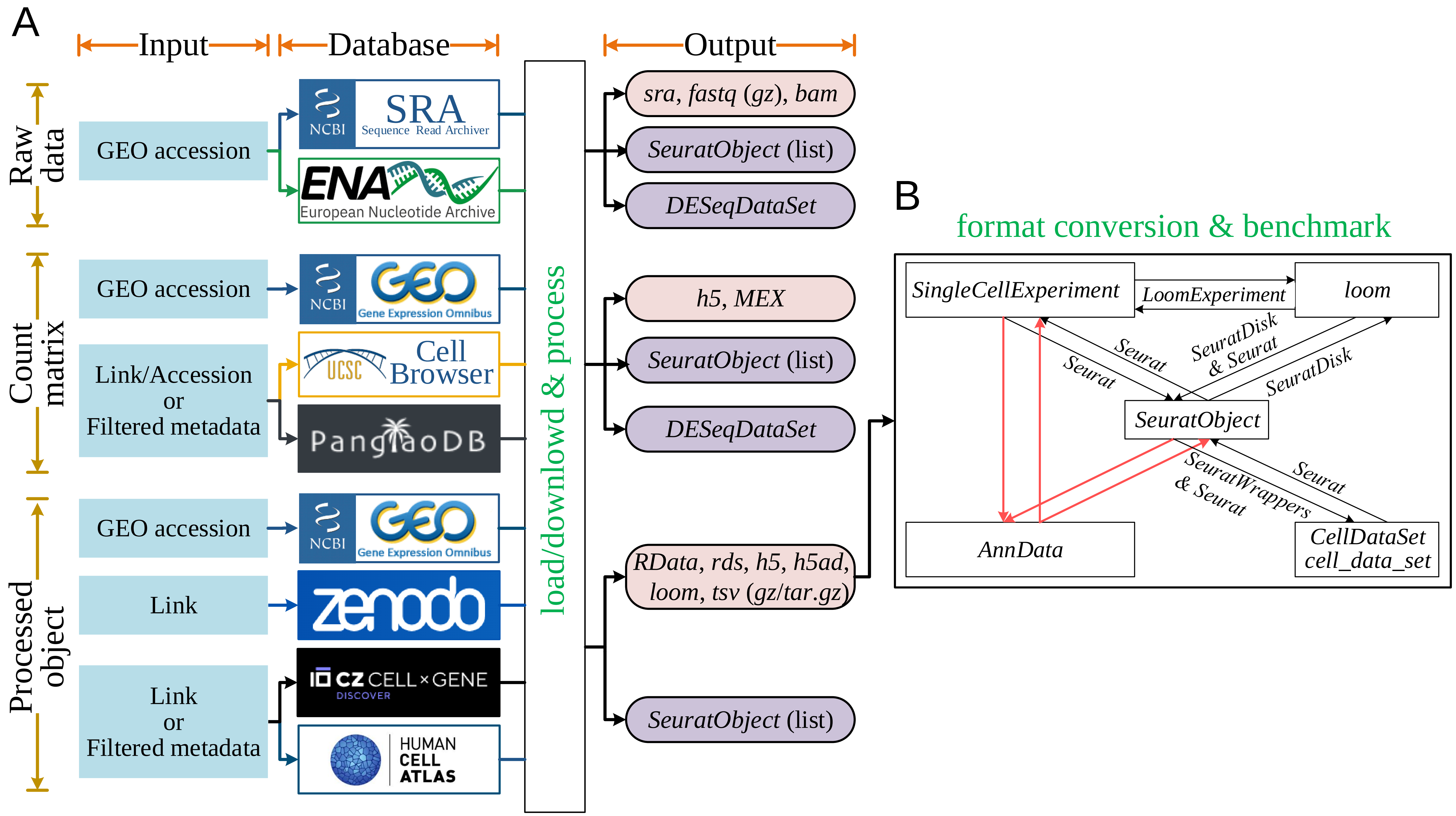

Click here to access/download;Figure;Figure 2.pdf

Figure 2 Application of GEfetch2R in COVID-19 scRNA-seq atlas exploration

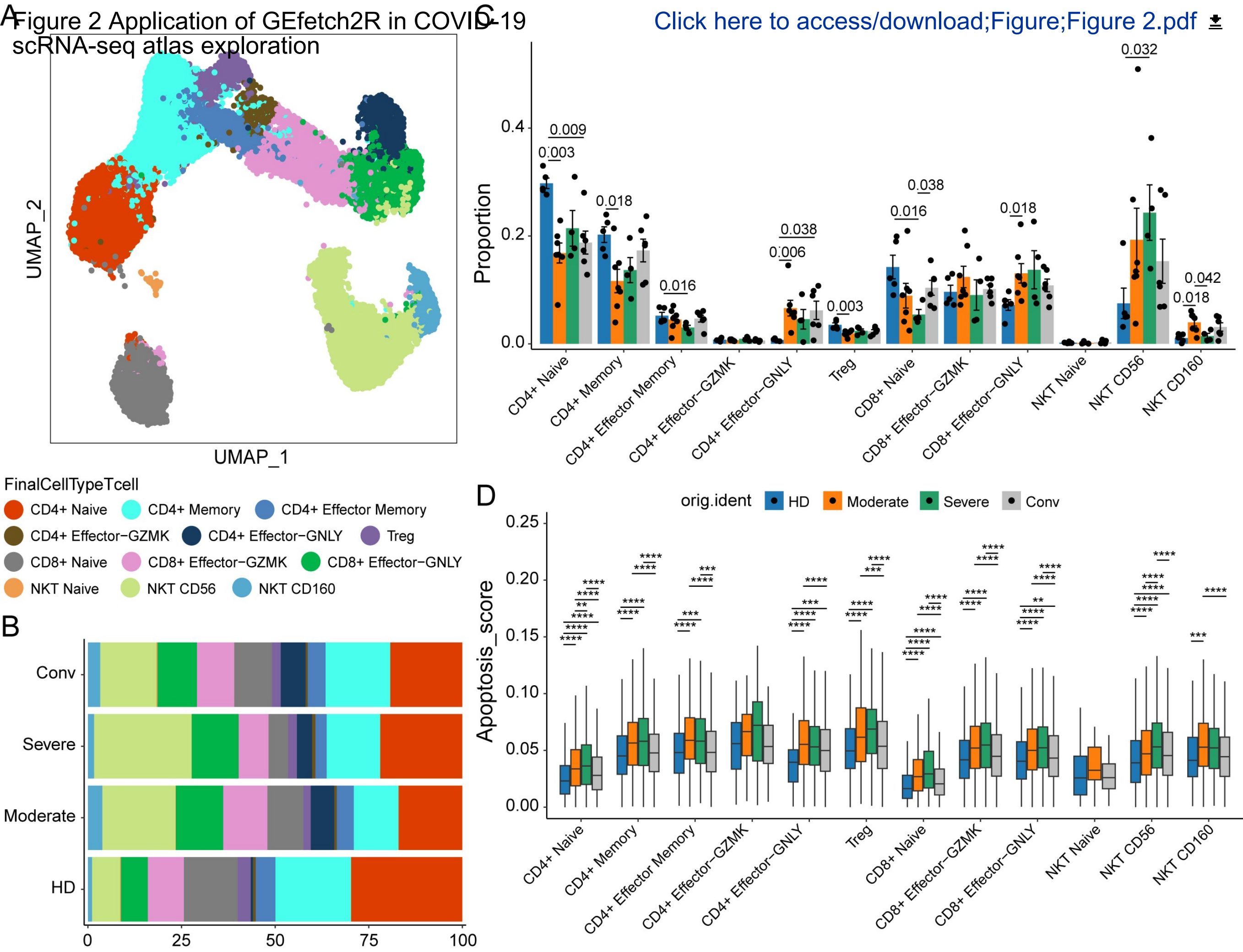

Click here to access/download;Figure;Figure 3.pdf

Figure 3 The information kept and usability of format conversion tools

A

Conversion	Tool	Count matrix	Annotation	Notes	Information rank
Seu2AD	SeuratDisk	data, counts/scale.data(conditional)	obs (meta.data), var (meta.features), uns, obsm (reductions), obsp (graphs), layers (assays)	the obsp only contains distances, not connectivities	2
	sceasy	counts/data/scale.data	obs (meta.data), var (meta.features), obsm (reductions)		3
	scDIOR	counts, data, scale.data(conditional)	obs (meta.data), var (meta.features), obsm (reductions), layers (assays), obsp (graphs)		1
SCE2AD	sceasy	counts/data/scale.data	obs (colData), var and varm (rowData), obsm (reducedDim)		3
	scDIOR	counts, data, scale.data	obs (colData), var (rowData), obsm (reducedDim), layers (assays)		2
	zellkonverter	counts, data, scale.data	obs (colData), var and varm(rowData), obsm (reducedDim), layers (assays), uns (metadata), obsp (colPairs)		1
AD2Seu	SeuratDisk	scale.data, counts/data(conditional)	meta.data (obs), meta.features (var), reductions (obsm, varm), misc (uns)	the misc is incomplete, only includes pca and umap	2
	sceasy	scale.data, counts/data(conditional)	meta.data (obs), meta.features (var), reductions (obsm)	the meta.features is incomplete	3
	scDIOR	scale.data, counts/data(conditional)	meta.data (obs), meta.features (var), reductions (obsm), assays (layers), graphs (obsp)	the meta.features is incomplete	1
	schard	counts/data/scale.data	meta.data (obs), meta.features (var), reductions (obsm)		4
AD2SCE	scDIOR	counts/data/scale.data	colData (obs), rowData (var), reducedDim (obsm), assays (layers)		2
	zellkonverter	scale.data, counts/data(conditional)	colData (obs), rowData (var, varm), reducedDim (obsm), assays (layers), metadata (uns), colPairs (obsp)		1
	schard	counts/data/scale.data	colData (obs), rowData (var), reducedDim (obsm)		3

B

Conversion	Tool	Programming language	Dependency	Availability	Documanetation	Support	Compatibility	Others	Usability rank
Seu2AD	SeuratDisk	R		GitHub, Conda	README, webpage	no Q&A	excellent	two steps, generate intermediate file	2
	sceasy	R	Python env	GitHub, Conda	README	no Q&A	excellent		3
	scDIOR	R, Python		GitHub	README	no Q&A	excellent	generate h5 instead of h5ad file	1
SCE2AD	sceasy	R	Python env	GitHub, Conda	README	no Q&A	excellent		3
	scDIOR	R, Python		GitHub	README	no Q&A	excellent	generate h5 instead of h5ad file	1
	zellkonverter	R	Python env	GitHub, Conda, Bioconductor	webpage	active Q&A	excellent		2
AD2Seu	SeuratDisk	R		GitHub, Conda	README, webpage	no Q&A	fail (failure on test and converted datasets)	two steps, generate intermediate file	3
	sceasy	R	Python env	GitHub, Conda	README	no Q&A	good (failure on test datasets, but success on zellkonverter converted datasets)		2
	scDIOR	R, Python	Python env	GitHub	README	no Q&A	fail (failure on test and converted datasets)		4
	schard	R		GitHub	README	active Q&A	excellent		1
AD2SCE	scDIOR	R, Python	Python env	GitHub	README	no Q&A	excellent		3
	zellkonverter	R	Python env	GitHub, Conda, Bioconductor	webpage	active Q&A	excellent		2
	schard	R		GitHub	README	active Q&A	excellent		1

Figure 4 The running time of format conversion tools

Click here to access/download;Figure;Figure 4.pdf

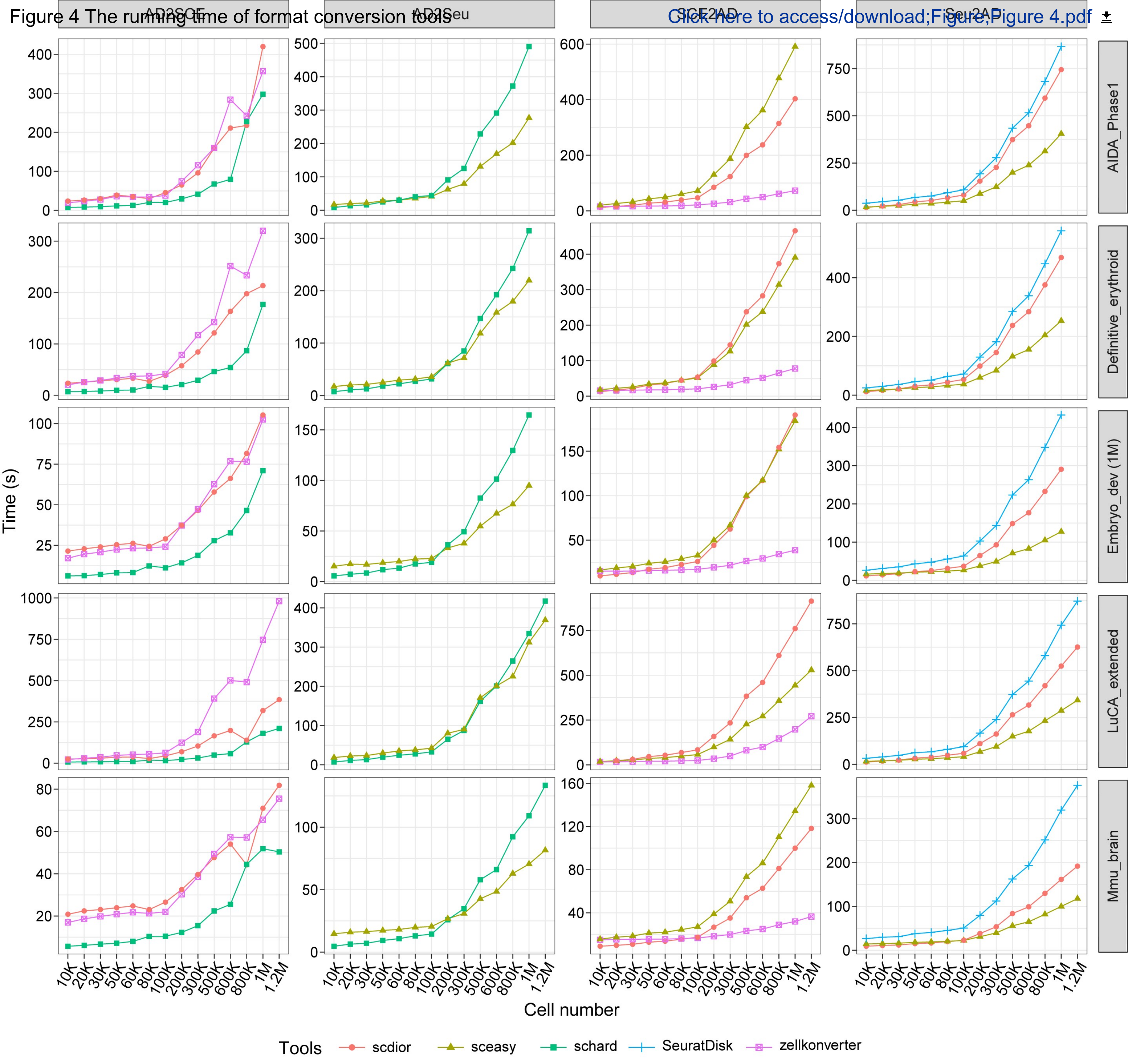

Supplementary Figure S1 The detailed workflow of GEfetch2R

Click here to access/download
Supplementary Material
Supplementary Figure S1.png

Supplementary Table S1 The datasets used for benchmarking format conversion tools

Click here to access/download
Supplementary Material
Supplementary Table S1.xlsx

Supplementary Table S2 The running time of the format conversion tools on five object sets

Click here to access/download
Supplementary Material
Supplementary Table S2.xlsx

Supplementary Note Step-by-step code and output showing information kept of format conversion tools

Click here to access/download
Supplementary Material
Supplementary Note.html
