# Supplementary material for: GEfetch2R: fetching single-cell/bulk RNA-seq data from public repositories to R and benchmarking the subsequent format conversion tools
Source: Gigascience. 2026 Apr 8;15:giag039. doi: 10.1093/gigascience/giag039 (PMC13147242; doi:10.1093/gigascience/giag039)
Supplement: giag039_Authors_Response_To_Reviewer_Comments_original_submission [file giag039_authors_response_to_reviewer_comments_original_submission.pdf]

Author's Response To Reviewer Comments

Replies to reviewers' comments (PDF version and Supplementary Data/Table can be accessed via https://github.com/showteeth/GEfetch2R/raw/refs/heads/main/man/benchmark/Response%20to%20Reviewers.zip):

>We thank all reviewers for taking the time to read our manuscript and for giving suggestions to improve it. We truly appreciate the positive assessment of our work. In this document, we attempt to address all concerns and questions raised by reviewers.

Reviewer #1: The manuscript presents GEfetch2R, an R package (with a Docker image) that fetches scRNA-seq and bulk RNA-seq data from multiple repositories, loads the data into R objects, and benchmarks format-conversion tools. The problem addressed is real and important; the implementation appears practical and well documented. I see strong potential for adoption.

>We are deeply grateful for your concise summary and your profound interest in our study.

Major comments
1. Robust cross-repository support for .RData files
While GEfetch2R lists rdata among supported extensions for Zenodo and HCA, many GEO submissions and other archives still provide processed data exclusively as .RData, often bundling matrices and metadata in heterogeneous objects. Please add an explicit, repository-agnostic .RData ingestion path with: (i) automatic object introspection, (ii) standardized extraction of matrices/metadata, (iii) graceful fallbacks with clear diagnostics for non-standard objects, and (iv) reproducible examples. This materially increases real-world coverage.

>In response to the reviewer's suggestion, we have made the following improvements: 1) added the function ParseGEOProcessed to parse the processed objects (rds (load into R automatically if it's SeuratObject), RData, loom, and h5ad) from GEO; 2) added the function LoadRData to dissect and extract the RData files. The LoadRData loads the RData file to a separate environment, distinguishes the class of each object available, and processes the objects according to the following logic (the detailed procedures are attached as Supplementary Data 1 (https://github.com/showteeth/GEfetch2R/raw/refs/heads/main/man/benchmark/Response%20to%20Reviewers.zip) and can be accessed via https://showteeth.github.io/GEfetch2R/articles/DownloadObjects.html#process-rdata-files):
* if widely used scRNA-seq objects (SeuratObject (Seurat package), seuratobject (Seurat v2 package), SingleCellExperiment (SingleCellExperiment package), cell_data_set (Monocle v3 package), CellDataSet (Monocle package)) or bulk RNA-seq objects (DESeqDataSet (DESeq2 package), DGEList (edgeR package)) exist, LoadRData automatically distinguishes the object class and extracts the raw count matrix, normalized count matrix, scaled count matrix, and metadata.
* else if non-standard objects (matrix, data.frame, dgCMatrix, dgRMatrix, dgTMatrix) exist, for matrix/data.frame objects, check the class of each column value and the name of every column, treat objects with numeric/integer values as count matrices, treat objects with sample group/annotation related column names as sample metadata, otherwise display the structure of the objects and load the objects to R for manually check; for dgCMatrix/dgRMatrix/dgTMatrix objects, treat them as count matrices directly.
* else print the first six (or less) elements of the objects.
>The cross-repository and reproducible examples are attached as Supplementary Data 1 (https://github.com/showteeth/GEfetch2R/raw/refs/heads/main/man/benchmark/Response%20to%20Reviewers.zip) and can be accessed via https://showteeth.github.io/GEfetch2R/articles/DownloadObjects.html#process-rdata-files. For more examples and evaluation results, please refer to the next response.

2. Large-scale, automated evaluation on ~100 scRNA-seq datasets
Beyond the single COVID-19 application and the conversion benchmark, please include a systematic "fetch success-rate" study across ~100 GEO scRNA-seq datasets. Provide a Dockerized workflow (publicly available) that periodically attempts end-to-end retrieval (raw / count / processed) and reports success/failure rates stratified by repository and file type, with resource/time footprints and categorized failure causes. Given heterogeneous deposition practices, even ~50% overall success would be informative.

>We agree that large-scale evaluation is crucial for testing the usability of GEfetch2R. However, a GEO accession could contain several supplementary files, which may include files unrelated to the count matrix. GEfetch2R usually requires users to specify a supplementary file (regardless of format, whether it's compressed or archived) as the file storing the count matrix. These bottlenecks make automated evaluation difficult. And as the second reviewer mentioned, users typically need GEfetch2R to access data for a specified accession. Thus, we conducted the large-scale evaluation by manually searching and selecting GEO accessions. It should be noted that this selection was performed before running GEfetch2R. The number of evaluation accessions selected for each category depends on the complexity of the supplementary files.
>The evaluation results, separated into "download" and "create R object", are attached as Table R1, Supplementary Data 2 and 3 (https://github.com/showteeth/GEfetch2R/raw/refs/heads/main/man/benchmark/Response%20to%20Reviewers.zip). In summary, for Smart-seq2 scRNA-seq, GEfetch2R supports loading count matrices from files in diverse formats (csv/tsv/txt/tab/xlsx/xls, whether compressed or/and archived, whether single file or multiple files) and files generated by various feature counting tools (STAR, htseq-count, featureCounts), removing preset or user-specified useless columns, automatically transposing the transposed count matrix (the number of rows/genes is less than the number of columns/samples), and creating the SeuratObject. The failure causes can be categorized as "file with comment lines", "first column isn't genes/features", and "unspecified numeric column". The success rates are 93.9% and 100%. For 10x Genomics scRNA-seq, GEfetch2R can create SeuratObjects from count matrices stored in h5 files (whether compressed or/and archived) and files in MEX format (whether compressed or/and archived). GEfetch2R also supports count matrices generated by scRNA-seq platforms other than 10x Genomics, which have a similar output structure (MEX format) to 10x Genomics, e.g., SeekOne and MobiDrop. The failure causes can be categorized as "incorrect file name", "missing file", "unsupported format", and "parameter adjustment". The success rates are 94.0% and 98.4%. GEfetch2R supports downloading processed objects in RData, rds, h5ad, and loom formats (whether compressed or/and archived), loading SeuratObjects stored in rds files to R, and dissecting and extracting the RData files. The success rates are 100% and 92.9%-100%. The failure cause is "key word misidentification" and the Seurat version is a risk factor. The overall success rates are 96.2% and 98.6%. The evaluation codes (GEO_evaluation_download.R, GEO_evaluation_createObject.R) are in the Docker image (soyabean/gefetch2r:1.2, path: /home/rstudio/GEOBench) and the GEfetch2R GitHub repository (https://github.com/showteeth/GEfetch2R/tree/main/man/benchmark).
>To further facilitate users in checking the usability of GEfetch2R, we have provided the function CheckAPI to automatically check the API availability of various databases (https://showteeth.github.io/GEfetch2R/articles/DownloadRaw.html#check-api, https://showteeth.github.io/GEfetch2R/articles/DownloadMatrices.html#check-api, https://showteeth.github.io/GEfetch2R/articles/DownloadObjects.html#check-api).

3. Another very important point is to provide a Dockerfile together with the Docker.

>Thanks for your reminder. We now provide the Dockerfile and necessary files (e.g. Docker_test.R: codes for testing the usability of the image; GEO_evaluation_download.R and GEO_evaluation_createObject.R: codes for conducting the large-scale evaluation) via GitHub (https://github.com/showteeth/GEfetch2R/tree/main/Docker/).

Minor revisions
"altas" → atlas (COVID-19 section title/caption).
"Count maatrix" → Count matrix (Figure 3 caption/table column).
"PanglanDB" → PanglaoDB (tables).
Consistency: keep SeuratObject (not "Seurat object"); keep rds lowercase;

>We apologize for the previous misspelling and inconsistency. All the above mistakes have been corrected in the revised manuscript (lines 270, 276, 291, 466; line 320; Table 1, Table 2, documentation, website; documentation, website). We also further checked the manuscript to avoid similar issues.

Reviewer #2: Every day, more than 30 single cell datasets are published. Almost none of them in a homogenous format, and not available anywhere but in obscure archives. Making the data more accessible is a major problem, which NIH and NBCI in particular bungled. They could have funded one group to make this easier or tell GEO to do it,

but they didn't. So now we're getting packages like this one here who spend years on fixing this problem with complex commands.
So the authors here provide yet another single cell downloader/processing package, but this one compares itself to the previous ones, and includes steps for aligning and for making the expression matrix. This type of software is really needed, with more than a dozen different single cell websites, it's annoying to convert data all the time.

>Many thanks for your precise summary of the current data acquisition status and your recognition in our study.

Major:
My main feedback is that the documentation is somewhat hard to find and that there is no "quick start" tutorial. The main question that the user who comes to your website has is "how do I import dataset X now" ? For that you need a docs page linked from the top of your Github where it walks the user through importing a dataset. Not finding it, because we can use Google for that. Once i have a link to a GEO dataset, or a cell browser dataset, how can I load the expression matrix? I think that should be covered. I needed to click around quite a bit to find this page: https://showteeth.github.io/GEfetch2R/articles/DownloadMatrices.html but it confuses the user by telling me how to download all datasets and finding the right one, but in most cases, I already have the right one. And this page should be easier to find.

>This is an excellent point. As suggested, we have added the "Quick start" tutorial in the GitHub README.md (https://github.com/showteeth/GEfetch2R?tab=readme-ov-file#quick-start-1) and in a new webpage of the GEfetch2R website (https://showteeth.github.io/GEfetch2R/articles/QuickStart.html). Besides, we have also added prominent links for users to conveniently access this tutorial at the very top of the GitHub README.md (https://github.com/showteeth/GEfetch2R?tab=readme-ov-file#quick-start) and the welcome page of the GEfetch2R website (https://showteeth.github.io/GEfetch2R/index.html).
>In the "Quick start" tutorial, we provide commands to access data based on the categorization of databases instead of data types, which makes it easier for users to retrieve data based on the database-specific links or accessions. For databases where the metadata of all datasets or projects are available (PanglaoDB, UCSC Cell Browser, CELLxGENE, and Human Cell Atlas), we classify data access strategies into two types: based on given accessions/links (e.g. https://showteeth.github.io/GEfetch2R/articles/QuickStart.html#given-dataset-1) and based on filtered metadata (e.g. https://showteeth.github.io/GEfetch2R/articles/QuickStart.html#filter-samples-based-on-metadata-1). For databases such as GEO and Zenodo, we provide the data access strategy based on given accessions or links (e.g. https://showteeth.github.io/GEfetch2R/articles/QuickStart.html#zenodo-processed-object). We hope that those strategies can simultaneously meet users' needs for downloading specified data and downloading data with similar characteristics in bulk.

Minor:
1. One database that you are missing entirely is Nemo analytics alias gEar, but that's totally OK. The manuscript is very complete and covers the main features and justifies its use of other packages, which in itself is already interesting.

>Thank you again for your supportive appraisal. The gEAR portal (https://umgear.org/dataset_explorer.html) is an amazing platform for data deposition, display, analysis and interrogation, with a focus on allowing users to customize the site and their own data displays. I have figured out the way to download the processed objects by providing shareable links. However, after reading the article and conducting a cursory search of issues on GitHub, I couldn't find an available API. I'm not sure if it's allowed to directly provide a download function (I have created an issue on GitHub: https://github.com/IGS/gEAR/issues/1176). If it is, we would be happy to offer such a function in the next release. In the revised manuscript (lines 420-421), I regard it as an important aspect for future improvement.

2. This page https://showteeth.github.io/GEfetch2R/articles/DownloadMatrices.html does not cover Cellxgene. I don't know how that's possible: you added Cellxgene support, but then forgot to document it?

>The CELLxGENE database provides processed objects (h5ad and rds) for downloading, so the tutorial can be found at https://showteeth.github.io/GEfetch2R/articles/DownloadObjects.html. To avoid possible confusion, we now list the corresponding databases alongside the tutorials (https://github.com/showteeth/GEfetch2R?tab=readme-ov-file#vignette, https://showteeth.github.io/GEfetch2R/index.html#vignette).

3. The https://showteeth.github.io/GEfetch2R/articles/DownloadRaw.html, just like the other page above, requires a

ton of settings. It would be nice if this was simpler. The docker container could set environment variables so I don't have to provide the directories for the SRA tools all the time. Right now, the package is far from easy to run. I understand that this is a difficult task, but some automated assistant, wrapper function, where I can just run downloadFileAndAlign(accession, genome) would be really nice. The website can be guessed from the accession and the 10x or not format can be guessed from the metadata.

>Following the reviewer's suggestion, we have added a wrapper function DownloadFastq2R to simplify the procedures from downloading data to loading into R, while minimizing the number of parameters. The DownloadFastq2R can extract all runs under a given accession, automatically identify the RNA-seq type (10x Genomics scRNA-seq, bulk RNA-seq, Smart-seq2 scRNA-seq/mini-bulk RNA-seq) of each run, download fastq files from ENA, perform read alignment and feature counting (merge multiple runs of a sample), and load the results to R (SeuratObject/DESeqDataSet). The DownloadFastq2R automatically identify the RNA-seq type according to the following logic:
* filter the library_strategy field ("rna-seq", "scrna-seq", "snrna-seq") to remove non-RNA-seq runs
* if library_source field is "transcriptomic single cell" or library_strategy field is in c("scrna-seq", "snrna-seq") or key words ("scRNA", "snRNA", "single.cell", "single.nuclei", "single.nucleus", "singlecell", "singlenuclei", "singlenucleus", "10X.Genomics", "10XGenomics", "Smart.seq2", "Smartseq2") appear in title|source_name|characteristics|description fields, the run is classified as scRNA-seq:
- if key words ("10X.Genomics", "Cell.Ranger", "10XGenomics", "CellRanger") appear in any field, the run is classified as 10x Genomics scRNA-seq
- if key words ("Smart.seq2", "Smartseq2") appear in any field, the run is classified as Smart-seq2 scRNA-seq/mini bulk RNA-seq
- else the run will skip subsequent processing steps
* else the run is classified as bulk RNA-seq
>If the above automatic identification failed, users can specify the RNA-seq type via parameter "force.type".
>The key parameters needed are GEO accession ("acce")/GSM accessions ("gsm"), path to the mapper ("star.path"/"cellranger.path", can be automatically detected), and path to the reference genome ("star.ref"/"cellranger.ref"). The examples can be found at https://showteeth.github.io/GEfetch2R/articles/DownloadRaw.html#one-step-wrapper.
>Additionally, we are committed to simplifying the data access procedures from other databases. Currently, users only need to provide links/accessions to retrieve count matrices or processed objects from PanglaoDB, UCSC Cell Browser, Zenodo, CELLxGENE, Human Cell Atlas.

4. With packages like this, more than the initial release, what's most important to get users is support and updates. I hope that even if the author is a grad student, that the lab's PI can continue supporting this package. Otherwise this risks just becoming another abandoned package.

>We agree that support and updates are crucial for the usability of GEfetch2R. The biggest factor affecting the functionality of GEfetch2R is the availability of the APIs. We briefly reviewed the GitHub commit records (the initial commit was on Jul 12, 2023), the APIs to access GEO, PanglaoDB, and UCSC Cell Browser are quite robust with no failures, and the APIs to access Zenodo, CELLxGENE, and Human Cell Atlas had minor changes in the past. To conveniently and quickly check the availability of APIs, we have added the function CheckAPI (https://showteeth.github.io/GEfetch2R/articles/DownloadRaw.html#check-api, https://showteeth.github.io/GEfetch2R/articles/DownloadMatrices.html#check-api, https://showteeth.github.io/GEfetch2R/articles/DownloadObjects.html#check-api).
>As a frequently used tool in our laboratory, we will ensure its ongoing support and timely updates in the future, as long as the continuous availability of database APIs. We believe that the publication of this tool will further ignite our enthusiasm and attract more users to provide feedback and suggestions for improvement, thereby refining the tool.

Cookie Preference Center

We use cookies which are necessary to make our site work. We may also use additional cookies to analyze, improve and personalize our content and your digital experience. For more information, see our Cookie Policy.

You may choose not to allow some types of cookies. However, blocking some types may impact your experience of our site and the services we are able to offer. See the different category headings below to find out more or change your settings.

Allow all

Manage Consent Preferences

Strictly Necessary Cookies

Always active

These cookies are necessary for the website to function and cannot be switched off in our systems. They are usually only set in response to actions made by you which amount to a request for services, such as setting your privacy preferences, logging in or filling in forms. You can set your browser to block or alert you about these cookies, but some parts of the site will not then work.

Functional Cookies

These cookies enable the website to provide enhanced functionality and personalisation. They may be set by us or by third party providers whose services we have added to our pages. If you do not allow these cookies then some or all of these services may not function properly.

Performance Cookies

These cookies allow us to count visits and traffic sources so we can measure and improve the performance of our site. They help us to know which pages are the most and least popular and see how visitors move around the site.

Targeting Cookies

These cookies may be set through our site by our advertising partners. They may be used by those companies to build a profile of your interests and show you relevant adverts on other sites. If you do not allow these cookies, you will experience less targeted advertising.

Cookie List

Clear

Apply Cancel
Consent Leg.Interest

Confirm my choices

Powe
