# Supplementary material for: GEfetch2R: fetching single-cell/bulk RNA-seq data from public repositories to R and benchmarking the subsequent format conversion tools
Source: Gigascience. 2026 Apr 8;15:giag039. doi: 10.1093/gigascience/giag039 (PMC13147242; doi:10.1093/gigascience/giag039)
Supplement: giag039_Reviewer_2_Report_original_submission — Reviewer 2 -- 12/3/2025 [file giag039_reviewer_2_report_original_submission.pdf]

Reviewer Report

Title: GEfetch2R: fetching single-cell/bulk RNA-seq data from public repositories to R and benchmarking the subsequent format conversion tools

Version: Original Submission **Date:** 12/3/2025

Reviewer name: Reviewer 2

Reviewer Comments to Author:

Every day, more than 30 single cell datasets are published. Almost none of them in a homogenous format, and not available anywhere but in obscure archives. Making the data more accessible is a major problem, which NIH and NBCI in particular bungled. They could have funded one group to make this easier or tell GEO to do it, but they didn't. So now we're getting packages like this one here who spend years on fixing this problem with complex commands.So the authors here provide yet another single cell downloader/processing package, but this one compares itself to the previous ones, and includes steps for aligning and for making the expression matrix. This type of software is really needed, with more than a dozen different single cell websites, it's annoying to convert data all the time.Major:My main feedback is that the documentation is somewhat hard to find and that there is no "quick start" tutorial. The main question that the user who comes to your website has is "how do I import dataset X now" ? For that you need a docs page linked from the top of your Github where it walks the user through importing a dataset. Not finding it, because we can use Google for that. Once i have a link to a GEO dataset, or a cell browser dataset, how can I load the expression matrix? I think that should be covered. I needed to click around quite a bit to find this page: https://showteeth.github.io/GEfetch2R/articles/DownloadMatrices.html but it confuses the user by telling me how to download all datasets and finding the right one, but in most cases, I already have the right one. And this page should be easier to find.Minor:One database that you are missing entirely is Nemo analytics alias gEar, but that's totally OK. The manuscript is very complete and covers the main features and justifies its use of other packages, which in itself is already interesting.This page https://showteeth.github.io/GEfetch2R/articles/DownloadMatrices.html does not cover Cellxgene. I don't know how that's possible: you added Cellxgene support, but then forgot to document it?The https://showteeth.github.io/GEfetch2R/articles/DownloadRaw.html, just like the other page above, requires a ton of settings. It would be nice if this was simpler. The docker container could set environment variables so I don't have to provide the directories for the SRA tools all the time. Right now, the package is far from easy to run. I understand that this is a difficult task, but some automated assistant, wrapper function, where I can just run downloadFileAndAlign(accession, genome) would be really nice. The website can be guessed from the accession and the 10x or not format can be guessed from the metadata.With packages like this, more than the initial release, what's most important to get users is support and updates. I hope that even if the author is a grad student, that the lab's PI can continue supporting this package. Otherwise this risks just becoming another abandoned package.

Level of Interest

Please indicate how interesting you found the manuscript: Choose an item.

Quality of Written English

Please indicate the quality of language in the manuscript: Choose an item.

Declaration of Competing Interests

Please complete a declaration of competing interests, considering the following questions:

- Have you in the past five years received reimbursements, fees, funding, or salary from an organisation that may in any way gain or lose financially from the publication of this manuscript, either now or in the future?
- Do you hold any stocks or shares in an organisation that may in any way gain or lose financially from the publication of this manuscript, either now or in the future?
- Do you hold or are you currently applying for any patents relating to the content of the manuscript?
- Have you received reimbursements, fees, funding, or salary from an organization that holds or has applied for patents relating to the content of the manuscript?
- Do you have any other financial competing interests?
- Do you have any non-financial competing interests in relation to this paper?

If you can answer no to all of the above, write 'I declare that I have no competing interests' below. If your reply is yes to any, please give details below.

I declare that I have no competing interests

I agree to the open peer review policy of the journal. I understand that my name will be included on my report to the authors and, if the manuscript is accepted for publication, my named report including any attachments I upload will be posted on the website along with the authors' responses. I agree for my report to be made available under an Open Access Creative Commons CC-BY license (http://creativecommons.org/licenses/by/4.0/). I understand that any comments which I do not wish to be included in my named report can be included as confidential comments to the editors, which will not be published.

Choose an item.

To further support our reviewers, we have joined with Publons, where you can gain additional credit to further highlight your hard work (see: https://publons.com/journal/530/gigascience). On publication of this paper, your review will be automatically added to Publons, you can then choose whether or not to claim your Publons credit. I understand this statement.

Yes Choose an item.
